# Integrating sign surveys and telemetry data for estimating brown bear (*Ursus arctos*) density in the Romanian Carpathians

**DOI:** 10.1002/ece3.3177

**Published:** 2017-08-01

**Authors:** Viorel D. Popescu, Ruben Iosif, Mihai I. Pop, Silviu Chiriac, George Bouroș, Brett J. Furnas

**Affiliations:** ^1^ Department of Biological Sciences Ohio University Athens OH USA; ^2^ Centre for Environmental Research (CCMESI) University of Bucharest Bucharest Romania; ^3^ Asociatia pentru Conservarea Diversitatii Biologice (ACDB) Focsani Romania; ^4^ Vrancea Environmental Protection Agency Focsani Romania; ^5^ California Department of Fish and Wildlife Wildlife Investigations Laboratory Rancho Cordova CA USA; ^6^ Department of Environmental Science, Policy and Management University of California Berkeley CA USA

**Keywords:** Carpathians, N‐mixture model, population density, Romania, track survey, *Ursus arctos*

## Abstract

Accurate population size estimates are important information for sustainable wildlife management. The Romanian Carpathians harbor the largest brown bear (*Ursus arctos*) population in Europe, yet current management relies on estimates of density that lack statistical oversight and ignore uncertainty deriving from track surveys. In this study, we investigate an alternative approach to estimate brown bear density using sign surveys along transects within a novel integration of occupancy models and home range methods. We performed repeated surveys along 2‐km segments of forest roads during three distinct seasons: spring 2011, fall‐winter 2011, and spring 2012, within three game management units and a Natura 2000 site. We estimated bears abundances along transects using the number of unique tracks observed per survey occasion via N‐mixture hierarchical models, which account for imperfect detection. To obtain brown bear densities, we combined these abundances with the effective sampling area of the transects, that is, estimated as a function of the median (± bootstrapped SE) of the core home range (5.58 ± 1.08 km^2^) based on telemetry data from 17 bears tracked for 1‐month periods overlapping our surveys windows. Our analyses yielded average brown bear densities (and 95% confidence intervals) for the three seasons of: 11.5 (7.8–15.3), 11.3 (7.4–15.2), and 12.4 (8.6–16.3) individuals/100 km^2^. Across game management units, mean densities ranged between 7.5 and 14.8 individuals/100 km^2^. Our method incorporates multiple sources of uncertainty (e.g., effective sampling area, imperfect detection) to estimate brown bear density, but the inference fundamentally relies on unmarked individuals only. While useful as a temporary approach to monitor brown bears, we urge implementing DNA capture–recapture methods regionally to inform brown bear management and recommend increasing resources for GPS collars to improve estimates of effective sampling area.

## 1 INTRODUCTION

Reliable population size and density estimates are important information for management or conservation actions aimed at ensuring the long‐term viability of wildlife populations (Ryman, Baccus, Reuterwall, & Smith, 1981). More specifically, large carnivores are generally cryptic animals often nocturnal and living in dense habitats (Linnell et al., 1998) that have large home ranges and tend to occur at low population densities. For these reasons, it is challenging to accurately estimate population size (Balme, Hunter, & Slotow, 2009; Ripple et al., 2014). Moreover, monitoring large carnivores to evaluate population size and trends often requires significant financial resources, which may be unavailable in certain regions, combined with large voluntary involvement (Dickman, Macdonald, & Macdonald, 2011; Kindberg, Ericsson, & Swenson, 2009), as well as access to state‐of‐the‐art technology, such as DNA‐based methods (DeYoung & Honeycutt, 2010).

Because large carnivores have been at the core of both intense management (e.g., through regulated hunting) and conservation initiatives (e.g., population recovery) for decades, a wide range of monitoring methodologies have been developed and implemented worldwide. Currently, the most reliable methods for estimating population size in carnivore populations are based on noninvasive genetic sampling (e.g., scat, hair, urine, saliva), in which individual detection histories can be used in a spatial capture–recapture framework (Borchers & Efford, 2008; Royle & Young, 2008). Camera trapping can also provide reliable inferences on population size for animals that can be uniquely identified (e.g., have unique patterns), especially when combined with DNA methods (e.g., Gopalaswamy et al., 2012; Royle, Nichols, Karanth, & Gopalaswamy, 2009). However, when animals cannot be uniquely identified, or when resources needed to perform genetic analyses are not available, low cost methods based on track and sign surveys are still largely used for monitoring carnivore populations (Lyra‐Jorge, Ciocheti, Pivello, & Meirelles, 2008; Wilson & Delahay, 2001). With adequate effort and application of statistical methods, track and sign methods can be effective for monitoring population changes in some large carnivore species (e.g., Eurasian lynx; Linnell et al., 2007). However, without statistical oversight, such methods may fail to detect population changes or may provide misleading estimates, thus hindering effective conservation and management of large carnivore populations (Popescu, Artelle, Pop, Manolache, & Rozylowicz, 2016).

The brown bear (*Ursus arctos*) is the largest carnivore species in Europe, and the populations have rebounded in the past decades in the European Union (Chapron et al., 2014), due to sustained conservation and management efforts. Brown bears are a species of conservation concern in Europe (listed in Annex IV of the Habitats Directive); EU member countries must ensure a Favorable Conservation Status within national boundaries, which requires reliable knowledge on population size and density (Trouwborst, Boitani, & Linnell, 2017). Brown bear densities have been estimated in many European countries using modern DNA‐based capture–recapture techniques, for example, Sweden (Bellemain, Swenson, Tallmon, Brunberg, & Taberlet, 2005; Kindberg et al., 2011), Slovenia (Jerina, Jonozovič, Krofel, & Skrbinšek, 2013), and Greece (Karamanlidis, de Gabriel Hernando, Krambokoukis, & Gimenez, 2015). Although Eastern European countries (including Russia; Bragina et al., 2015) have large carnivore populations distributed across broad geographic regions, they lack financial resources for monitoring. Thus, these countries are lagging behind in terms of implementing noninvasive genetic methods. Romania is one such country and is reported to harbor the largest brown bear population in Europe (outside European Russia; Rozylowicz, Popescu, Pătroescu, & Chişamera, 2011; Salvatori et al., 2002). Brown bear population monitoring in Romania relies on a mixture of track surveys and sightings at feeding stations by local wildlife managers; these data are pooled together by the national wildlife authorities yearly to estimate the total number of brown bears at county level (Cazacu et al., 2014). However, these monitoring methods ignore uncertainty, which compounded with the lack of statistical oversight, may yield unrealistically high population estimates (Popescu et al., 2016). Moreover, unsustainable quotas can trigger local declines of populations if they are implemented for long periods of time (Artelle et al., 2013; Packer et al., 2011).

To meet deficiencies in large carnivore monitoring in Romania, a consortium of state agencies and environmental NGO's implemented project LIFE08NAT/RO/000500–LIFEURSUS “Best practices and demonstrative actions for the conservation of *Ursus arctos* populations in Central‐Eastern Carpathians” (2010–2013), financed by the LIFE program, the EU's financial instrument supporting environmental, nature conservation, and climate action (http://ec.europa.eu/environment/life/). The project targeted the improvement of brown bear management and monitoring techniques applied in Romania. Specifically, the goal of this study was to build on current monitoring methods for game species implemented by the Romanian wildlife authorities and evaluate an alternative approach to estimate brown bear density at the level of game management unit, which integrates repeated track surveys and home range methods. Integrating different data types is an increasingly common practice in wildlife studies, as it leads to improved inferences on animal populations (e.g., Besbeas, Freeman, Morgan, & Catchpole, 2002; Furnas, Landers, Callas, & Matthews, 2017; Gopalaswamy et al., 2012; Ivan, White, & Shenk, 2013; Sollmann et al., 2013). In this study, we used repeated track counts to estimate abundances (at transect level) and independent home range information based on GPS telemetry data to calculate brown bear densities. We combined uncertainties from these two sources of information using the Delta method (Link & Nichols, 1994). The specific objectives of the study were as follows: (1) to quantify brown bear density at the level of game management unit by integrating sign data and independently gathered home range data, and (2) to identify the best predictors for brown bear abundance and detection probability from repeated sign surveys for two distinct survey periods (pre‐ and posthibernation).

## 2 MATERIALS AND METHODS

### 2.1 Study area

**Figure 1:**
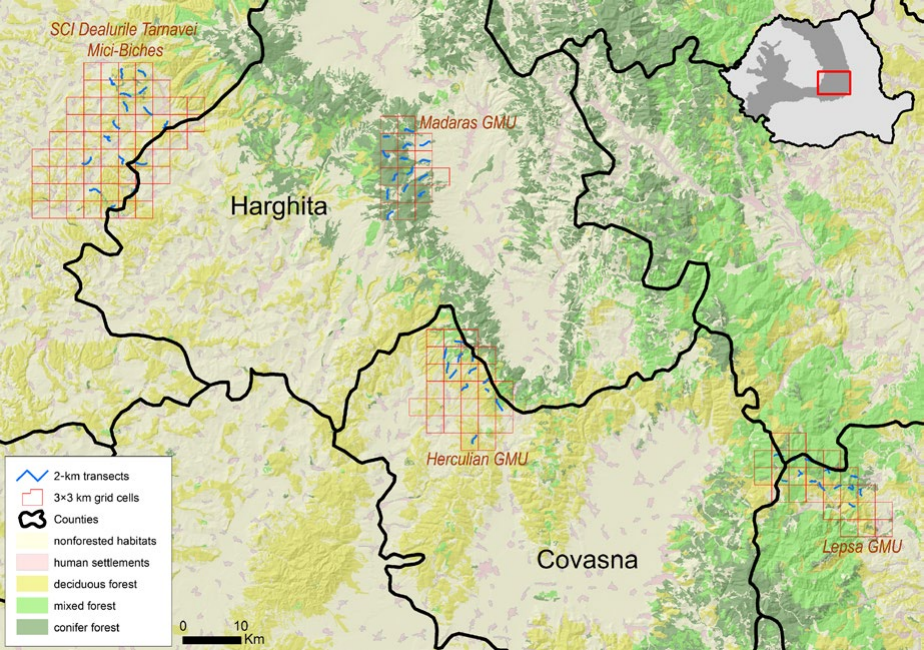
The study area is comprised of three game management units (GMU) and one Natura 2000 Site of Community Importance (SCI Dealurile Tarnavei Mici‐Biches) in the Romanian Carpathians. The Site of Community Importance partly overlaps on five game management units that were surveyed only in the last season (Mar‐Apr 2012). The four sites were divided in 3 × 3 km grid cells, within each grid cell we identified a 2‐km section of forest road, which served as the survey transect for bear tracks

The study area, located in the South of the Romanian Eastern Carpathians, was represented by three game management units (hereafter *GMU*) Lepsa—with an area of 111 km^2^, Herculian—167 km^2^, and Madaras—105 km^2^; in 2012, we additionally surveyed a Natura 2000 Site of Community Importance with an area of 379 km^2^, that is, SCI Dealurile Tarnavei Mici—Biches (hereafter called *Tarnave SCI*; Figure 1). The Romanian Carpathians harbor some of Europe's largest stands of old growth forests, and the three selected GMUs (Lepsa, Herculian, and Madaras) are largely forested (>85%). Forest composition is dominated by mixed forests of beech‐fir or beech‐fir‐spruce (*Fagus sylvatica*,* Abies alba,* and *Picea abies*), with *Fagus spp*. dominating at lower elevations (600–700 m), and coniferous species at higher elevations (1,100–1,300 m). Tarnave SCI it is located at lower elevation (600 m average) and has a heterogeneous mixed deciduous forests and nonforested habitats composition. The large mammal community is intact throughout the study area, and composed of the three European large carnivores: wolf (*Canis lupus*), brown bear (*Ursus arctos*), Eurasian lynx (*Lynx lynx*), as well as ungulates: wild boar (*Sus scrofa*), roe deer (*Capreolus capreolus*), and red deer (*Cervus elaphus*). The road network within the GMUs is poor and dominated by unpaved forestry roads and temporary logging roads. Logging is the main economic activity in the study area and continues year round, with higher intensity between October and May. The period with consistent snow pack varies with altitude, but it overall ranges from November–December to April–May.

### 2.2 Track surveys

The study area was first divided in 3 × 3 km grid cells, and within each grid cell, we selected a 2‐km section of forest/logging road, which served as the surveys transect for bear tracks (Figure 1). The extent of both cells and transects was selected based on existing information on brown bear movement ecology in the study area from an independent dataset (from previous large carnivore LIFE Nature projects implemented in Eastern Carpathians; www.carnivoremari.ro). Specifically, VHF and GPS telemetry data indicated that, in our study region, the average daily distance traveled by brown bears during the pre‐ and posthibernation period was 1.5 km (www.carnivoremari.ro). Based on this information, the 2‐km transects were separated by >1.5 km, which would minimize the chances of double counting the same bear on different transects, given that adjacent transects are surveyed within the same day or within 36 hr. We conducted repeated snow and mud track sign surveys during three distinct seasons pre‐ and posthibernation: March–April 2011 (three repeat surveys on 27 transects in three GMUs), November–December 2011 (four repeat surveys on 36 transects in three GMUs), and March–April 2012 (four repeat surveys on 49 transects in three GMUs and Tarnave SCI). Pre‐and posthibernation represents a period of relatively low activity for brown bears in Romanian Carpathians, and all repeat surveys within a season were performed within a short, 30‐day period in an attempt to meet the population closure assumption.

We collected four types of biometric data on all brown bear tracks detected: width and length of anterior and posterior foot track. Surveys were mostly performed after fresh snowfall to ensure that only fresh tracks were counted, and that track measurements were not impacted by snowmelt. The data collection on transects within each GMU was performed in a 36‐hr interval in order to reduce the chance of double counting the same animal on different transects. To minimize the likelihood that we counted the same animal multiple times on a given transect and survey period, we discarded all tracks older than 24–48 hr, as well as all 24‐ to 48‐hr‐old tracks without precise measurements. Additionally, we observed other track characteristics, such as movement direction and track configuration (based on topography or habitat structure), and discounted any possible “double counting” tracks as a first filter (i.e., tracks of similar size that repeatedly occur, e.g., in one direction on the same valley). Finally, to convert track measurements to unique number of individuals, we eliminated the tracks with differences between measurements <2–3 cm identified on a given transect at a given survey time. For example, if three tracks or sets of tracks were identified on a given transect during one survey and their lengths were front length 1 = 24 cm, front length 2 = 22 cm, front length 3 = 25 cm, similar differences for the rest of the measurements, we assumed that they belonged to the same individual, and therefore, the estimate for the given transect for that survey was 1 individual (e.g., see Appendix S1). Fresh tracks with differences in measurements >4–5 cm were assumed to belong to different individuals. The resulting dataset thus estimated a minimum number of individuals that intersected each transect during each survey, avoiding “double counting” of the same bear on a particular transect (see Table 1 for the frequency of successful detection of tracks on transects, and Appendix S2 for the final dataset used in the analysis).

**Table 1:** Survey occasions with at least one track identified

	Seasons			
	March–April 2011	November–December 2011	March–April 2012	
No. of detections	55	41	87	
No. of nondetections	25	96	94	
% detections	68.7	29.9	48.1	

	Sites			
	Herculian GMU	Lepsa GMU	Madaras GMU	Tarnave SCI
No. of detections	46	33	58	43
No. of nondetections	104	71	77	21
% detections	40.3	33.1	46.0	67.2

### 2.3 Statistical analyses

#### 2.3.1 Abundance modeling

We created a history of the track data representing a minimum number of individuals detected per transect per visit. Brown bears are a solitary, nonterritorial carnivore species (McLellan & Hovey, 2001); thus, our sampling likely met the assumption that detections of different individuals were independent. Assuming independence among detections based on both the species ecology and the sampling design, we used the N‐mixture method for count data (Royle, 2004), which relies on the robust design proposed by Pollock (1982) and Kendall, Nichols, and Hines (1997). This method accounts for imperfect detection when estimating abundance per sampling unit by integrating two processes: a state process (animal abundance per transect) and an observation process conditional upon the state of the site. Specifically, due to low track counts per occasion, the state process can be modeled as a Poisson process:

(1) $$N_{i}∼Poisson\left(\lambda_{i}\right),$$

where λ_*i*_ is the expected abundance at site *i*.

The observation process conditional on the abundance is:

(2) $$y_{it}∼Binomial\left(N_{i},p_{it}\right),$$

where *y*_*ij*_ is the number of distinct individuals counted at location *i* at time *t*,* N*_*i*_ is the number of individuals available for sampling at site *i*, and *p* is the probability of detecting an individual at location *i* during survey visit *t*.

We ran separate sets of models for each sampling season. We modeled imperfect detection using survey‐specific covariates: snow depth [*Snow*] and substrate [*Substrate = Snow, Mud, Dry*] as probability of detecting the bear tracks varies according to substrate properties, and Julian day [*Julian*] to account for the variation in probability of detection over the surveys duration. We also added a first‐order Markov process reflecting whether (1) or not (0) any bears were detected in the previous survey visit (*auto*; Hines et al., 2010; Slauson, Baldwin, & Zielinski, 2012; Sweitzer, Furnas, Barrett, Purcell, & Thompson, 2016). We modeled abundance per transect using site‐specific covariates: GMUs and the Natura 2000 SCI [*Site = Madaras, Lepsa, Herculian, Tarnave*] to address differences in abundance determined by local latent conditions; dominant forest type within 1‐km buffers around transects [*Dominant = Conifer, Deciduous, Mixed*] and percent of forest cover within the same buffer (*Conifer*,* Mixed*,* Deciduous*) as habitat‐type variables; and mean elevation of transect [*Altit*] as a proxy for overwintering climate conditions (higher elevations have longer snowpack). The land cover covariates were extracted from the 2006 Corine Land Cover dataset (European Environment Agency, Copenhagen, Denmark). Covariates were included into the N‐mixture models through *log* (site‐specific covariates for the state process) and *logit* (survey‐specific covariates for the observation process) functions.

We first ran models to identify the best covariates predicting detection for each season separately, using a global model and 11 combinations of detection covariates that were determined a priori (Appendix S3). After identifying the best detection covariates for each season, we ran 16 models (including the *Null* model) selected a priori for estimating abundance of tracks per transect (Appendix S3). We used AICc (Akaike Information Criterion) to rank the models and performed model averaging to estimate abundance per transect from the full set of models and to identify the best predictors for brown bear abundance (Burnham & Anderson, 2002). We ran the models in program R 3.2.3 (R Core Team, 2015) using function *pcount* in package *unmarked* (Fiske & Chandler, 2011), which fits the N‐mixture occupancy models (Royle, 2004). We summarized the model‐averaged abundance estimates into mean number of bears per transect (and 95% confidence intervals) for each season and GMU.

#### 2.3.2 Brown bear density

One of the challenges for quantifying animal abundance from sign surveys is that the effective sampling area is often unknown. We accounted for uncertainty in brown bear movement during the sampling windows by combining our abundance results with auxiliary information on home range size to estimate density within the study area.

We used GPS telemetry data from 17 bears (six females and 11 males) collected within several studies across the Eastern Romanian Carpathians between 2004 and 2015 and extracted telemetry locations recorded during seasons corresponding to our surveys: 15 November–15 December and 20 March–30 April. We used the telemetry data to build home ranges for each animal using fixed kernel density method implemented in *adehabitat* package for R program (Calenge, 2006). We used h_ref_ bandwidth estimation method for the kernel because it was documented to work properly with spatially clustered relocations that lack long‐distance movements (i.e., as in the case of our dataset selected for the two‐one‐month periods; see Appendix S4 and see Walter, Fischer, VerCauteren, & Baruch‐Mordo, 2011 for decisions on selecting bandwidth methods according with species movement patterns). For each individual home range, we extracted the core (50% isopleth) to be used in subsequent analyses, as the probability of detection is proportional to the frequency of space use at any location within the home range (i.e., the utilization distribution; Popescu, de Valpine, & Sweitzer, 2014); thus, movements within the core home range are more likely to be captured by transect surveys compared to sparser movements at the home range periphery. We used individuals which had minimum 30 fixes (Seaman et al., 1999) within the two‐one‐month sampling periods.

To calculate brown bear densities, we first calculated radii of circles equal in area to the median of the core home range size. We decided to use the median instead of the mean to minimize the influence of outliers as the distribution of home range areas was strongly right skewed. To get an estimate of standard error as well as upper and lower 95% confidence intervals, we used a bootstrap procedure extracting 10,000 random samples with replacement such that the size of each resample was equal to the original sample. We used these radii to build buffers around the 2‐km transects, resulting in a range of effective areas sampled during our surveys (denominator in equation 3). We then divided the model‐averaged predictions of abundance per transect derived from track data by the median of the effective area based on home ranges to get an estimate of density (equation 3). This approach was suggested to be the basis for density calculations in case of mammals (Dice, 1938). We calculated the variance around the density estimate by combining uncertainty in both abundance per transect and effective sampled area estimates using the Delta method (Link & Nichols, 1994; Powell, 2007) (Appendix S3).

(3) $$d=100\times \frac{\text{abund}}{\text{hr}\;+\;2\;\times \;\text{t.length}\;\times \;\sqrt{\frac{\text{hr}}{\pi }}}\;,$$

where *d* = density (individuals/100 km^2^); abund* =* model‐averaged abundance per transect, hr is the median of the 50% isopleth home range area, and t.length is the transect length.

**Figure 2:**
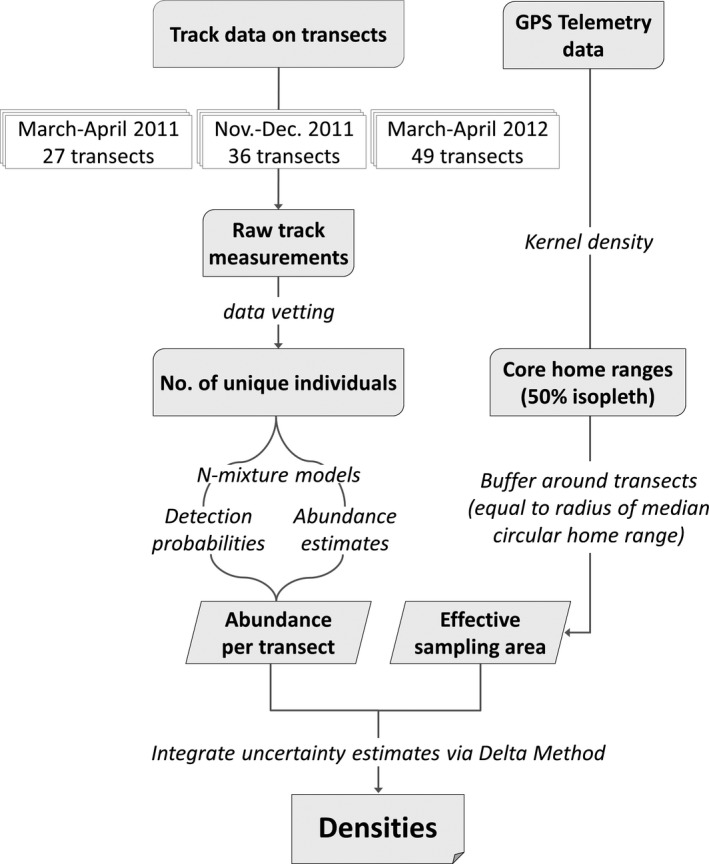
Flowchart summarizing the steps of our modeling approach to estimate brown bear densities in the Romanian Carpathians by combining track data on transects with GPS telemetry resources

This procedure allows one to approximate the sampling variance of a derived parameter (i.e., density) constructed as a function of other parameters that already have defined variance (i.e., abundance, home range size). A complete R script used to estimate densities is available in Appendix S3, and a flowchart summarizing the steps of our modeling approach is available in Figure 2.

## 3 RESULTS

### 3.1 Track surveys

Within transect, track measurements indicated a maximum of two different‐sized tracks or individuals per survey occasion. This led us to a total of 202 different tracks during the entire study period. We did not record any tracks on 25 transects of 112 revisits, six in March–April 2011, nine in November–December 2011, and 10 in March–April 2012. The probability of detecting tracks varied across seasons as follows: average detection probability (±SE) was 0.478 ± 0.115 in March–April 2011, 0.264 ± 0.104 in November–December 2011, and 0.363 ± 0.105 in March–April 2012. Moreover, November–December 2011 had the lowest detection success, with 29.9% of survey occasions yielding at least one bear track, while 68.7% of occasions yielded tracks in March–April 2011 (Table 1). The success of detecting tracks also varied by site; Lepsa had 33.1% of the survey occasions with at least one bear track, Herculian had 40.3%, Madaras had 46.0%, and Tarnave SCI, which was surveyed only during the last season, had 67.2%.

### 3.2 Home range estimates

Median (±bootstrapped SE) of the core home range areas that we used in the density calculations was 5.58 ± 1.08 km^2^ (estimated for 17 bears across November–December and March–April). The bootstrapped lower and upper 95% confidence interval bounds used for density calculations were 2.7 and 7.3 km^2^ (Appendix S4). In general, the home range estimates showed high variability between individual bears, independent on the number of fixes used (e.g., the smallest territory size of 0.23 km^2^ from 692 telemetry fixes while largest territory size of 61.85 km^2^ from 626 telemetry fixes; Appendix S4).

### 3.3 Brown bear density

The estimated average abundance of individuals per transect based on track measurements was consistent across the three seasons (i.e., 1.264 ± 0.094, 1.235 ± 0.117, and 1.363 ± 0.069 individuals per transect) and varied across sites (Table 2). Average brown bear densities (and 95% confidence intervals) derived from estimates of abundance per transect and home range estimates were similar across the three seasons: 11.5 (7.8–15.3) individuals/100 km^2^ in March–April 2011, 11.3 (7.4–15.2) individuals/100 km^2^ in November–December 2011, and only slightly higher, 12.4 (8.6–16.3) individuals/100 km^2^ in March–April 2012 (Table 2). Across GMUs, the average densities varied between 7.5 individuals/100 km^2^ (i.e., Herculian in November–December 2011), and 14.8 individuals/100 km^2^ (i.e., Madaras in March–April 2011). Tarnave SCI had the highest density, of 14.7 (9.6–19.7 individuals/100 km^2^), while Madaras had the highest overall brown bear density across the three seasons: 13.7 (9.1–18.0 individuals/100 km^2^); however, given the relatively wide confidence intervals, there were no significant differences in brown bear density between sites (Table 3).

**Table 2:** Model‐averaged brown bear abundance per transect (±SE) derived from track counts using N‐mixture models for three distinct sampling seasons

Site	March–April 2011	November–December 2011	March–April 2012	Average abundance (bears/transect)
	Season 1	Season 2	Season 3	
Herculian GMU	1.096 ± 0.132	0.826 ± 0.145	1.392 ± 0.149	1.104 ± 0.082 95% CI = 0.943–1.265
Lepsa GMU	1.073 ± 0.141	1.324 ± 0.217	1.170 ± 0.130	1.189 ± 0.096 95% CI = 0.999–1.378
Madaras GMU	1.623 ± 0.206	1.557 ± 0.237	1.283 ± 0.124	1.487 ± 0.112 95% CI = 1.267–1.708
Tarnave SCI			1.608 ± 0.149	
Average abundance (bears/transect)	1.264 ± 0.094 95% CI = 1.079–1.448	1.235 ± 0.117 95% CI = 1.005–1.466	1.363 ± 0.069 95% CI = 1.227–1.499	

**Table 3:** Brown bear densities in individuals per 100 km^2^, and 95% confidence intervals (in parentheses) estimated from occupancy‐based model‐averaged abundances per transect, and 50% kernel home range information from telemetry data on 17 bears in Romanian Carpathians. To represent the effective sampling area, we used the median of the 50% kernel home range estimated for the periods of the track sign surveys (November–December and April–May)

Site	Area (km^2^)	March–April 2011	November–December 2011	March–April 2012	Average density (bears/100 km^2^)
		Season 1	Season 2	Season 3	
Herculian GMU	163.9	10.0 (6.2–13.8)	7.5 (4.1–10.9)	12.7 (8.1–17.3)	10.1 (6.8–13.4)
Lepsa GMU	110.6	9.8 (6.0–13.6)	12.1 (6.8–17.3)	10.7 (6.8–14.6)	10.8 (7.2–14.5)
Madaras GMU	117.7	14.8 (9.1–20.5)	14.2 (8.3–20.2)	11.7 (7.6–15.8)	13.7 (9.1–18.0)
Tarnave SCI	370.8			14.7 (9.6–19.7)	14.7 (9.6–19.7)
Average density (bears/100 km^2^)		11.5 (7.8–15.3)	11.3 (7.4–15.2)	12.4 (8.6–16.3)	

### 3.4 Predictors for detection and abundance per transect

Overall, all variables used to predict abundance per transect had low predictive power across all three seasons. The best variable for brown bear track detection was *snow depth* for March–April 2011 and November–December 2011, which was inversely related to detection rate. For these two seasons, highest detection was recorded when show depth ranged between 1 and 10 cm. Best detection variable for March–April 2012 was *substrate* (highest detection recorded when substrate = *Snow*).

**Figure 3:**
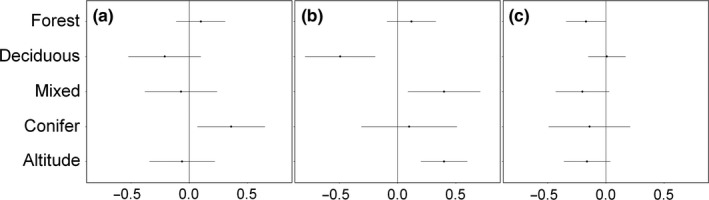
Standardized model‐averaged estimate coefficients ± unconditional standard error corresponding to five continuous covariates used to explain brown bear abundance for: (a)—March–April 2011, (b)—November–December 2011, (c)—March–April 2012

**Figure 4:**
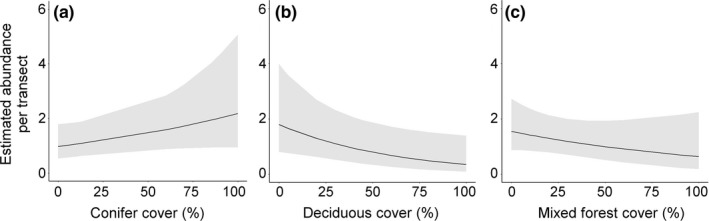
Brown bear estimated abundance per transect in response to the covariate of the best model for: (a)—March–April 2011, (b)—November–December 2011, (c)—March–April 2012

Following model averaging, we found that percent conifer cover within a 1‐km buffer around transects had a positive effect on abundance in March–April 2011 (AICwt = 0.178), percent deciduous forest cover had a negative effect on abundance in November–December 2011 (AICwt = 0.218), and percent mixed forest cover had a negative effect on abundance in March–April 2011 (AICwt = 0.133; Appendix S5; Figures 3 and 4).

## 4 DISCUSSION

Our study provides an alternative noninvasive method for estimating brown bear density at the scale of game management units in the Romanian Carpathians. By combining analyses of track sign to estimate abundance per transect and home range information to estimate the effective area of transects, our study demonstrates a more accurate way to estimate density that includes confidence intervals reflecting uncertainty from both data sources. Our method for estimating density during the pre‐ and posthibernation periods yielded relatively constant values across the three sampling seasons, despite variation across the four sites sampled within each season (Table 3). Because brown bear populations do not vary widely within the several‐month interval between sampling periods, this finding indicates that our approach is robust, and that monitoring during pre‐ and posthibernation seasons is likely to yield similar results. As such, this approach has the potential to be implemented at broad spatial scales with relatively low effort to produce brown bear density estimates that can be fine‐tuned at regular intervals using more robust (yet, logistically and financially intensive) methods such as genetic sampling. The novelty of our method consists in integrating common space use data (home range estimates) from independent datasets with survey data gathered at specific locations (transects). Using telemetry data specific to our 1‐month survey windows, gathered in the same general study area, we determined a biologically meaningful range of effective sampling area sizes (quantity often unknown in many wildlife studies) and used it to calculate densities based on track signs of unmarked individuals gathered on transects. As space use data becomes more readily available for many species, this general approach for estimating population density can be applied to other types of wildlife data, such as camera trap data (e.g., Furnas et al., 2017).

While our approach integrated different types of data for better inference, the densities estimated in our study are likely to provide an incomplete picture of the brown bear population in the area. While track surveys are effective in detecting the adult populations of large carnivores when the snow cover is still present (Linnell et al., 2007), surveying during the pre‐ and posthibernation seasons, when snow cover is adequate and bears restrict their movements have a lower chance of detecting females with cubs of the year or yearlings. Females with cubs can enter hibernation earlier and also abandon the denning site posthibernation later than adult females without cubs or males. Opportunistic observations as well as surveys targeted at identifying females with cubs during 2010–2013 in the study area suggested an increasing activity for females with yearlings in early May and for females with cubs of the year in late May–early June (Pop, Popescu, Chiriac, & Sandu, 2013). Specifically, Pop et al. (2013) identified between one and four females with cubs (yearlings or cubs of the year) per GMU between our three track survey windows. While the proportion of the females with cubs of the total population has not been quantified in Eastern and Central Europe, data from the other European populations suggests that it can be highly variable (Palomero et al., 2007). For example, Solberg, Bellemain, Drageset, Taberlet, and Swenson (2006) found that in Sweden, the proportion of females with yearlings or cubs of the year was 12.1% of the total population in 1 year and 42.0% in the second year of the study. The confidence intervals for brown bear densities in our study area (between seven and 16 individuals/100 km^2^ without females with cubs) are within the range of densities described elsewhere in Central and Eastern Europe although originated from different methodologies. In Slovenia, Jerina et al. (2013) merged four types of spatial distribution data (observations at feeding sites, removal data, GPS telemetry, and genetic samples) and obtained average densities of 13 individuals/100 km^2^ in the Dinaric Mountains (locally reaching 40 individuals/100 km^2^). In Slovakia, Rigg and Adamec (2007) merged direct observations with snow tracking and obtained values between five and 11 individuals/100 km^2^ while in Greece, Karamanlidis et al. (2015) used systematic genetic sampling and obtained densities of 1–5.4 individuals/100 km^2^.

### 4.1 Predictors of abundance

Overall, the variables used to model abundance per transect using track signs and N‐mixture models (Royle, 2004) had low explanatory power (Appendix S5; Figures 3 and 4). This was expected as the covariates used for modeling abundance across the study sites had low variability (e.g., percent cover of various forest types is rather constant within a 1‐km buffer around transects extracted from the 2006 Corine Land Cover dataset, with the exception of the last season, when mixed landscape of Tarnave SCI was included in the model). The abundance per transect was best predicted by percent conifer cover within a 1‐km buffer around transects in Season 1 (March–April 2011), suggesting a slight increase in abundance with increasing coniferous cover. In Season 2 (November–December 2011), bears abundance was negatively related to percent deciduous forest and positively related to mixed forest cover, but also by altitude. These relations are likely explained by the fact that bears move to higher grounds in search of remote areas for denning (e.g., Goldstein, Poe, Suring, Nielson, & McDonald, 2010). In the last season, abundance per transect was negatively related to forest cover in general, a direct result of including Tarnave SCI in the analysis, where nonforest habitats prevail. Overall, the lack of consistency in the variables predicting abundance across the three seasons are suggestive of brown bear as a generalist habitat species, which uses both mixed and coniferous forests for denning sites in our study area (S. Chiriac, unpublished data).

The most important source of uncertainty that affects the precision of our analysis was identifying the effective sampling area around each transect. This is a common issue in studies of unmarked animals, such as camera trapping or sign surveys, where extrapolating from abundances recorded at specific locations (camera sites or transects) to actual densities can be problematic. Here, we used the best available telemetry data for the larger Eastern Carpathian area to identify biologically meaningful effective sampling area. We added complexity to our approach by acknowledging variation in the spatial ecology of brown bears in the Eastern Carpathians during the three‐one‐month sampling windows. The median (±bootstrapped SE) of our 50% kernel home range estimate was 5.58 ± 1.08 km^2^ and matched the track survey periods of November–December and March–April. Thus, we aimed to capture the variability in the size of the territory used during pre‐ and posthibernation periods when bears movement is reduced, and they are retreating to remote and undisturbed denning sites (Schoen, Beier, Lentfer, & Johnson, 1987). We consider that the range of core areas used here (2.7–7.3 km^2^) captures the variation in movements during the pre‐ and posthibernation periods well. Moreover, our choice of using core areas (up to 50% isopleth) over the full 95% fixed kernel estimate is warranted by prior research that showed that the number of detections for carnivores is proportional to the utilization distribution (Popescu et al., 2014). In other words, it is likely that the greatest number of sign detections per sampling occasion per transect (up to 2) occurred in core areas rather than home range periphery. Yet, the density estimates are sensitive to the size of the effective sampling area and other studies may consider using a different home range estimates (and bandwidth method) to derive the effective sampling area around transects in respect with the research question in mind and species ecology. For example, Walter et al. (2011) suggest that Brownian Bridge Movement Models are a better representation of home range for organisms covering large areas, particularly migratory species, while Kernel Density methods are better suited for less mobile species.

Another source of uncertainty is related to the number of distinct tracks identified per sampling occasion per transect; because we used only fresh tracks on snow or mud (<24–48 hr) the measurements were less subject to bias, and we likely avoided double counting. However, it is possible that constraining the dataset to fresh tracks also eliminated some individuals, thus providing a conservative estimate of the number of tracks available for analysis. We acknowledge difficulties of discriminating between individuals with similar foot sizes (particularly subadults). Yet, we alleviated this source of bias in the field by assigning tracks to unique individuals not only based on their size, but also by examining track characteristics (such as orientation according to topography, direction of movement).

### 4.2 Management and conservation implications

Reliable abundance estimates that incorporate sources of uncertainty are essential to sound wildlife management and conservation. Specifically, for carnivore populations subject to regulated hunting, overoptimistic estimates of abundance can result in unsustainable harvest and compromise the long‐term viability of populations (Artelle et al., 2013). Our results corroborate the findings of Popescu et al. (2016) on the unrealism of brown bear population sizes provided by wildlife managers in Romania, which could be partially driven by hunting profitability. Carnivore trophy hunting has been banned in Romania in October 2016, due to concerns regarding the quality of data used in management and because the general public perceived ongoing carnivore trophy hunting practices as socially unacceptable or unsustainable. While the length of the trophy hunting ban is yet to be determined, this situation presents a unique opportunity to reassess current monitoring practices and promote the implementation of broadly accepted genotyping methods for determining carnivore densities (Mowat & Strobeck, 2000).

Applied broadly across many GMUs, the monitoring method described here has the potential to provide a better assessment of the population trend in the short term compared to current methods. However, the limitations of our study (e.g., missing females with cubs when surveying during optimal ground cover conditions), and additional sources of uncertainty, such as effective sampling area, and the resulting wide confidence intervals around density estimates, make the case for our method as a temporary solution. DNA‐based capture–recapture methods are currently used worldwide for evaluating brown bear densities, including in Central and Eastern Europe (Slovenia: Skrbinšek et al., 2012; Greece: Karamanlidis et al., 2015). Implementing such studies periodically (e.g., every 3–5 years) in Romania with an emphasis on areas with high levels of hunting (e.g., Eastern Carpathians), while they require considerable financial support, can provide the much needed benchmark against which our results, as well as official data, can be measured. A combination of the two approaches—repeated track counts and noninvasive genetic sampling—could be developed into a hybrid long‐term monitoring protocol (e.g., track‐based monitoring in between the years with genetic monitoring), as combining data types into single analysis frameworks provides improved inference on wildlife populations (Sollmann et al., 2013). Lastly, as most of the uncertainty affecting the precision of density estimates is due to uncertainties in the effective sampling area, it is recommended to increase resources for GPS collars and provide better coordination of GPS tracking efforts with field surveys (Furnas et al., 2017). An additional benefit of GPS collar data is that they could be combined with DNA data to improve the precision of spatial capture–recapture designs (Royle, Chandler, Sollmann, & Gardner, 2014), although implementing such designs at regional scale may exceed the financial possibilities of management agencies in developing countries.

## 5 CONCLUSIONS

Our study provides a first statistical estimate of brown bear density in the Romanian Carpathians based on tracking data, a traditional monitoring method used in Romanian wildlife management. These results are particularly important because they provide managers with more objective population parameters based on the data they have at hand, which could enable Romanian wildlife authorities to take informed and biologically meaningful management decision in the future. However, the multiple sources of uncertainty that have to be incorporated in the approach described here (from counting tracks to accounting for imperfect detection and determining home range sizes) highlight the need for reassessing the current monitoring practices and implementing modern and more robust monitoring methods, such as DNA‐based capture–recapture supported by GPS collars for estimating effective sampling area of any survey method.

## CONFLICT OF INTEREST

Authors have no conflict of interest to declare.

## AUTHOR CONTRIBUTIONS

VDP and MIP designed the study; MIP, SC, GB led data acquisition campaigns and performed data management; RI, VDP, and BJF analyzed the data and interpreted the results; VDP, RI, MIP, and BJF wrote the manuscript.
