# Supplementary material for: Integrating sign surveys and telemetry data for estimating brown bear (Ursus arctos) density in the Romanian Carpathians
Source: Ecol Evol. 2017 Aug 1;7(18):7134–44. doi: 10.1002/ece3.3177 (PMC5606905; doi:10.1002/ece3.3177)
Supplement: Supplementary file 1 [file ECE3-7-7134-s001.docx]

**Appendix S1.** Example converting raw track data to number of unique individuals per transect per survey occasion based on four biometric measurements

| **Track** | **Width anterior (cm)** | **Length anterior (cm)** | **Width posterior (cm)** | **Length anterior (cm)** | **Individual** |
| --- | --- | --- | --- | --- | --- |
| 1 | *14* | *12* | *13.5* | *24.5* | **bear 1** |
| 2 | *14.5* | *14* | *13.5* | *25* |  |
| 3 | *11* | *12* | *11* | *21.5* | **bear 2** |
| 4 | *12* | *12* | *12* | *20* |  |
