# Supplementary material for: Integrating sign surveys and telemetry data for estimating brown bear (Ursus arctos) density in the Romanian Carpathians
Source: Ecol Evol. 2017 Aug 1;7(18):7134–44. doi: 10.1002/ece3.3177 (PMC5606905; doi:10.1002/ece3.3177)
Supplement: Supplementary file 4 [file ECE3-7-7134-s004.docx]

**Appendix S4.** Home range estimates using fixed kernel method and extracted as 50% isopleth. We used GPS telemetry data from 17 bears collected across the Romanian Carpathians between 2004 and 2015 and extracted telemetry locations recorded during windows corresponding to our track surveys seasons: 15 November–15 December, and 20 March–30 April. We calculated median, standard error, lower and upper 95% confidence interval bounds using a bootstrap procedure with 10,000 random sampling replacements. Below we plotted the corresponding kernel utilization distributions in grayscale, 50% isopleth as a black line and relocations used for estimations in red.

| ID | Bear name (season) | No. of fixes | Sex | Core Home Range Area (km^2^) |
| --- | --- | --- | --- | --- |
| 1 | n1 (spring) | 131 | M | 48.74 |
| 2 | n11 (winter1) | 188 | F | 1.76 |
|  | n11 (spring1) | 143 |  | 0.33 |
|  | n11 (winter2) | 559 |  | 2.47 |
|  | n11 (spring2) | 765 |  | 54.38 |
| 3 | n19 (winter) | 30 | M | 5.81 |
|  | n19 (spring) | 67 |  | 4.27 |
| 4 | n2 (spring) | 32 | F | 7.66 |
| 5 | n3 (winter) | 57 | M | 6.30 |
| 6 | n4 (winter) | 66 | F | 0.98 |
|  | n4 (spring) | 60 |  | 0.48 |
| 7 | n5 (winter) | 162 | M | 6.08 |
|  | n5 (spring) | 125 |  | 30.32 |
| 8 | n9 (winter) | 608 | M | 7.21 |
|  | n9 (spring) | 701 |  | 7.42 |
| 9 | nl16 (winter) | 626 | M | 61.85 |
|  | nl16 (spring) | 489 |  | 7.24 |
| 10 | nl17 (winter) | 544 | M | 5.61 |
|  | nl17 (spring) | 429 |  | 8.18 |
| 11 | nr12 (winter) | 692 | F | 0.23 |
|  | nr12 (spring) | 749 |  | 0.97 |
| 12 | nR14 (winter) | 432 | M | 4.34 |
| 13 | nR18 (winter) | 266 | M | 2.44 |
|  | nR18 (spring) | 251 |  | 0.47 |
| 14 | nR20 (winter) | 65 | F | 5.51 |
| 15 | nR21(winter) | 47 | M | 3.06 |
| 16 | nR22(winter) | 83 | F | 17.10 |
| 17 | nR23(winter) | 64 | M | 36.80 |
|  |  |  |  | **Median = 5.58**  **se = 1.08**  **cv = 0.19**  **ci.lo = 2.77**  **ci.up = 7.33** |

n1 (spring)

n11 (winter1)

n11 (spring1)

n11 (winter2)

n11 (spring2)


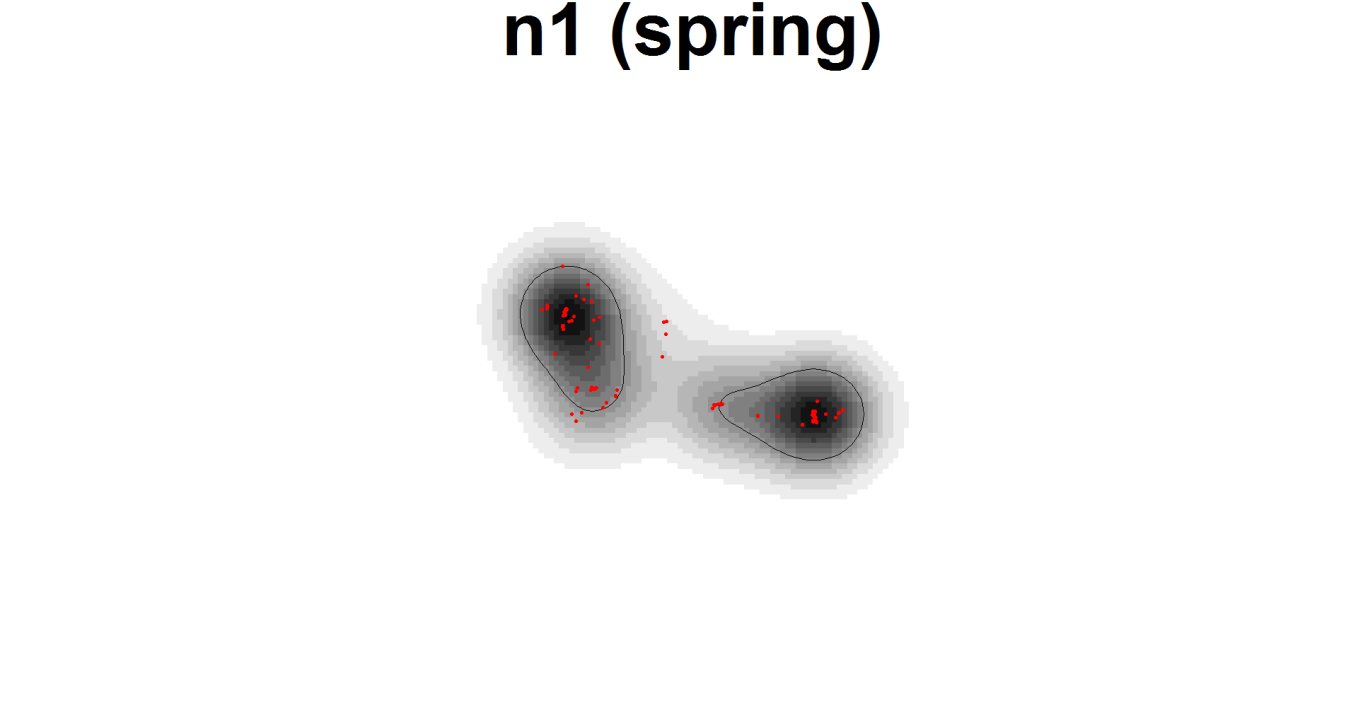

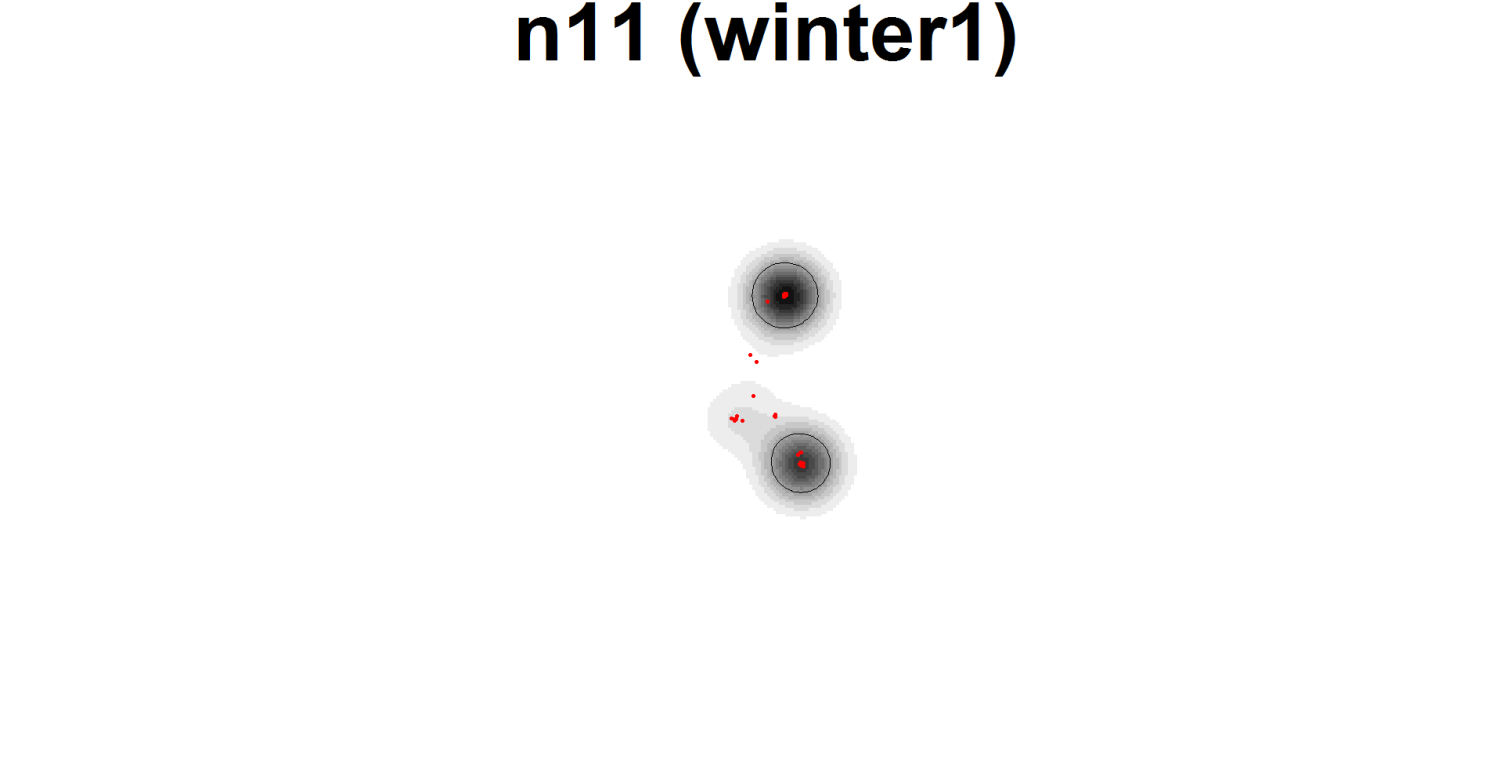

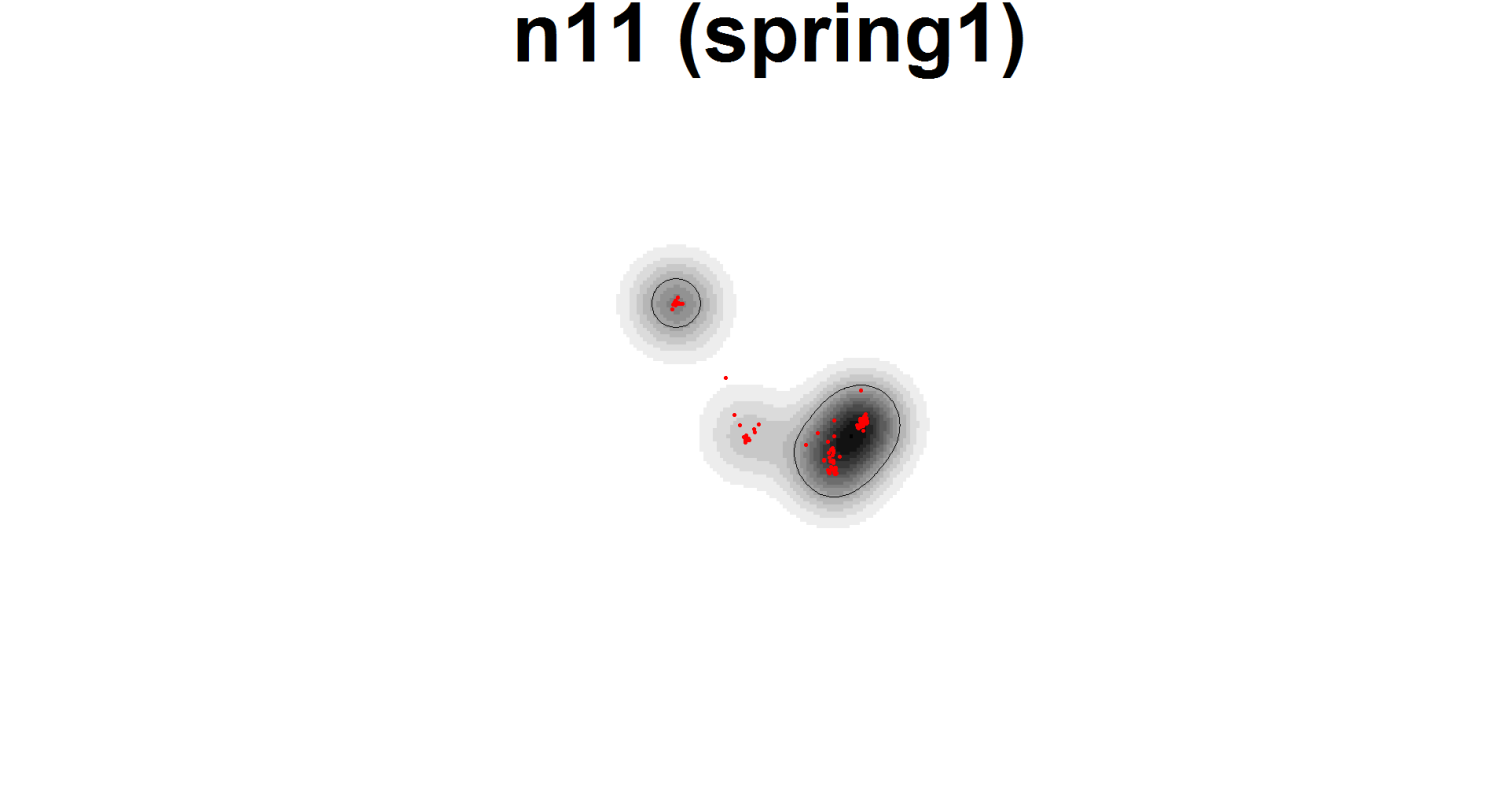

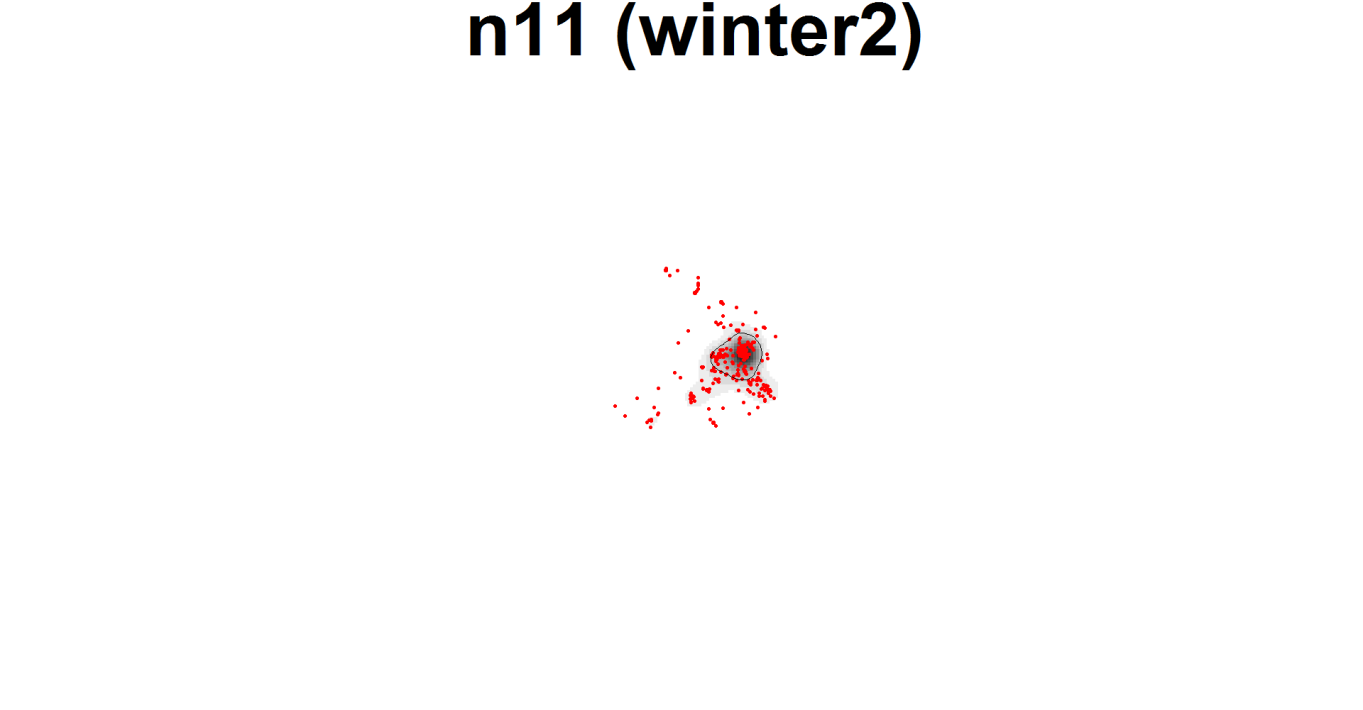

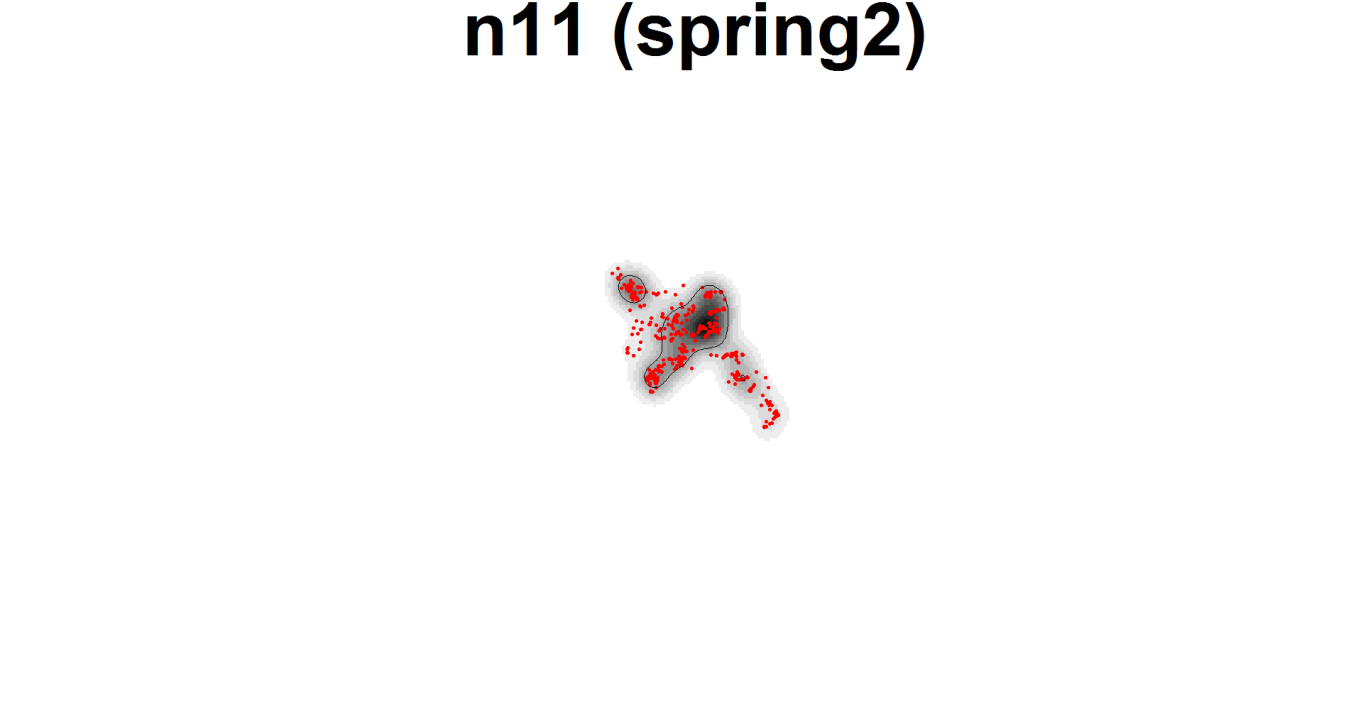

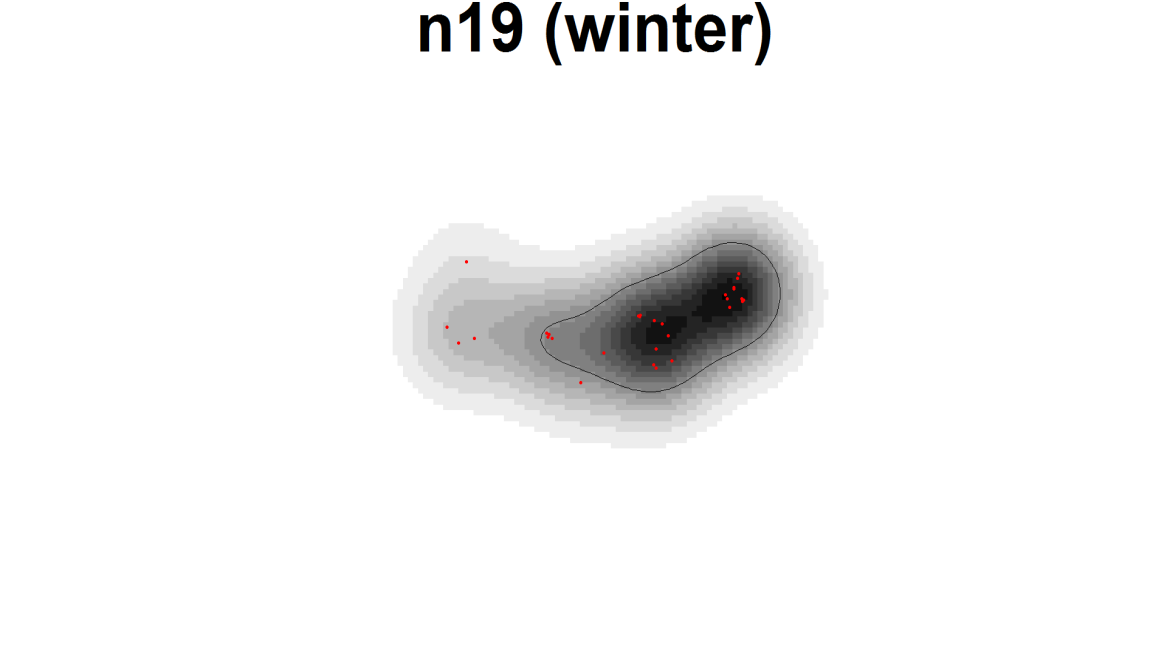


n19 (winter)

n19 (spring)

n2 (spring)

n3 (winter)


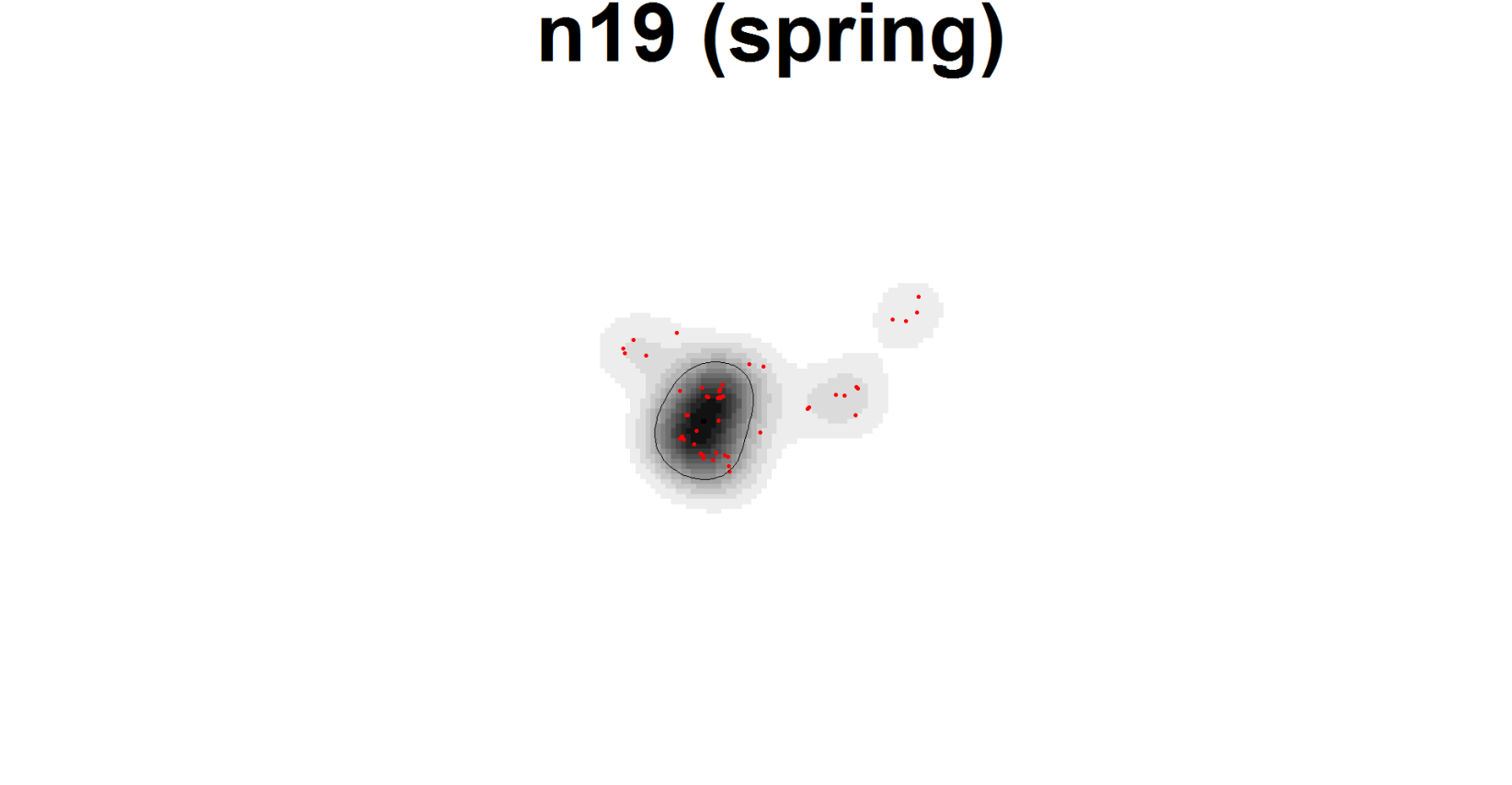

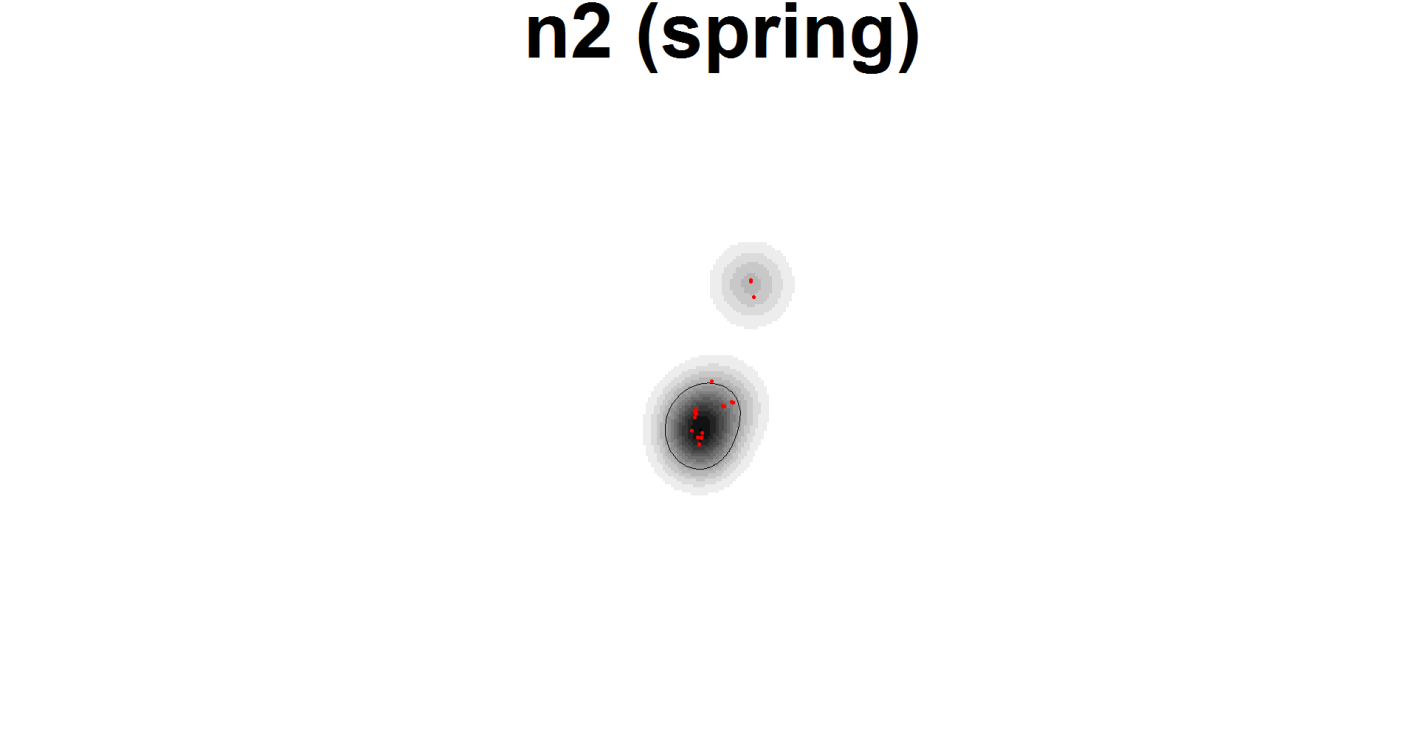

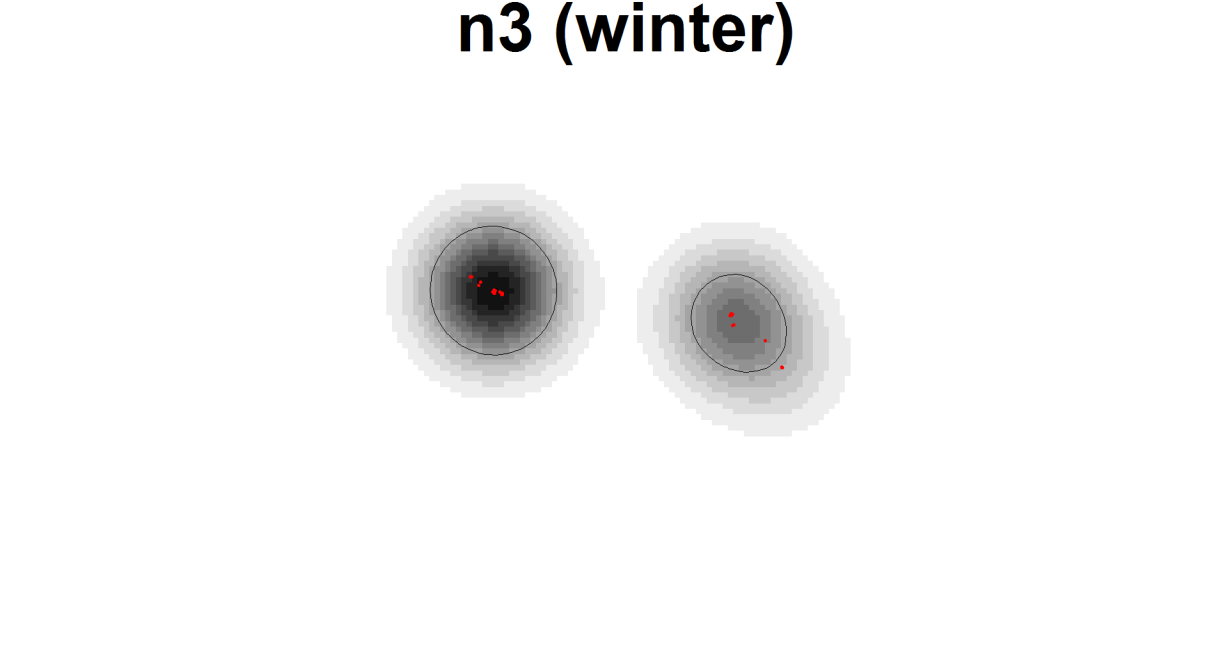

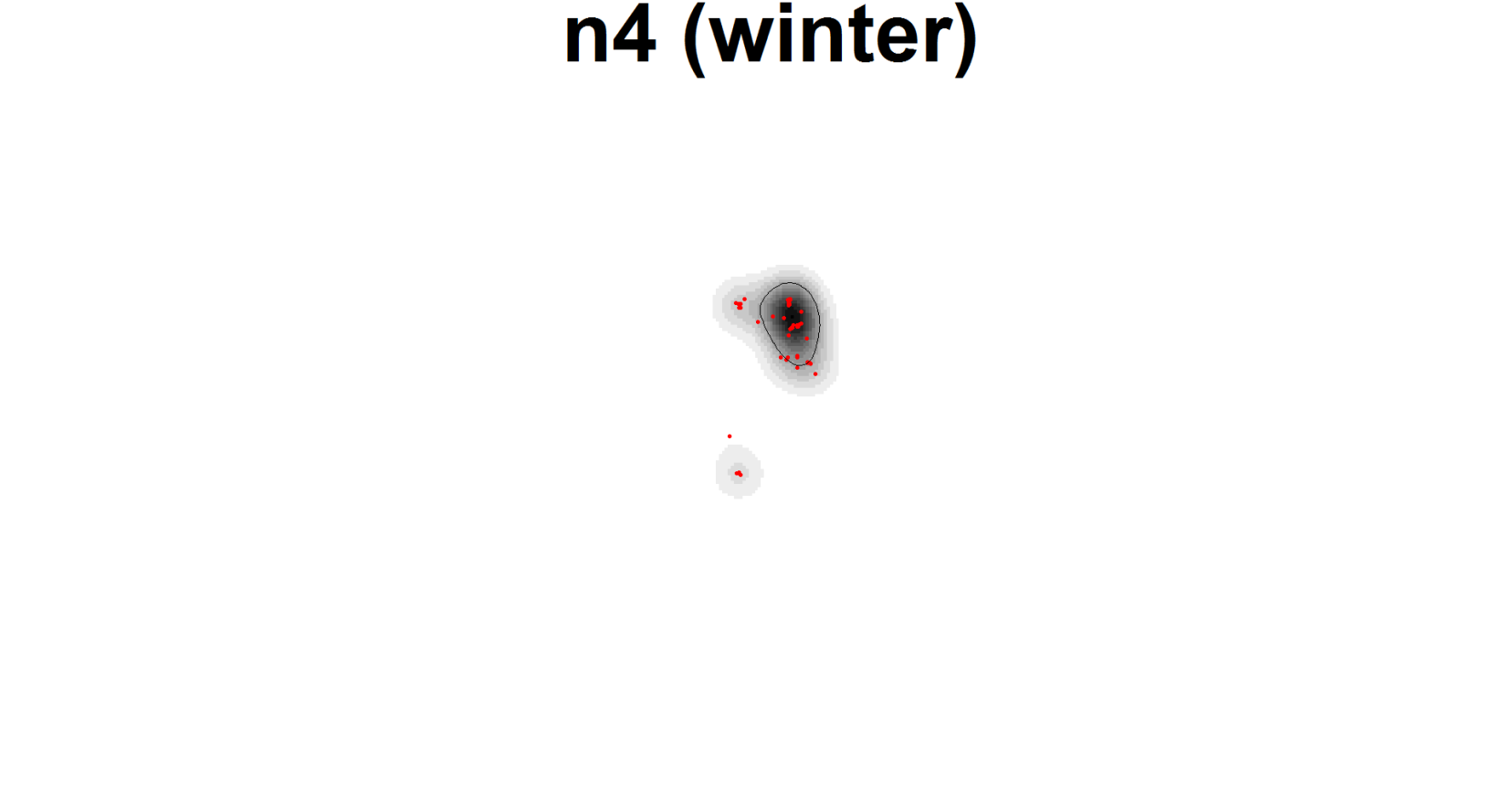


n4 (winter)

n4 (spring)

n5 (winter)

n5 (spring)


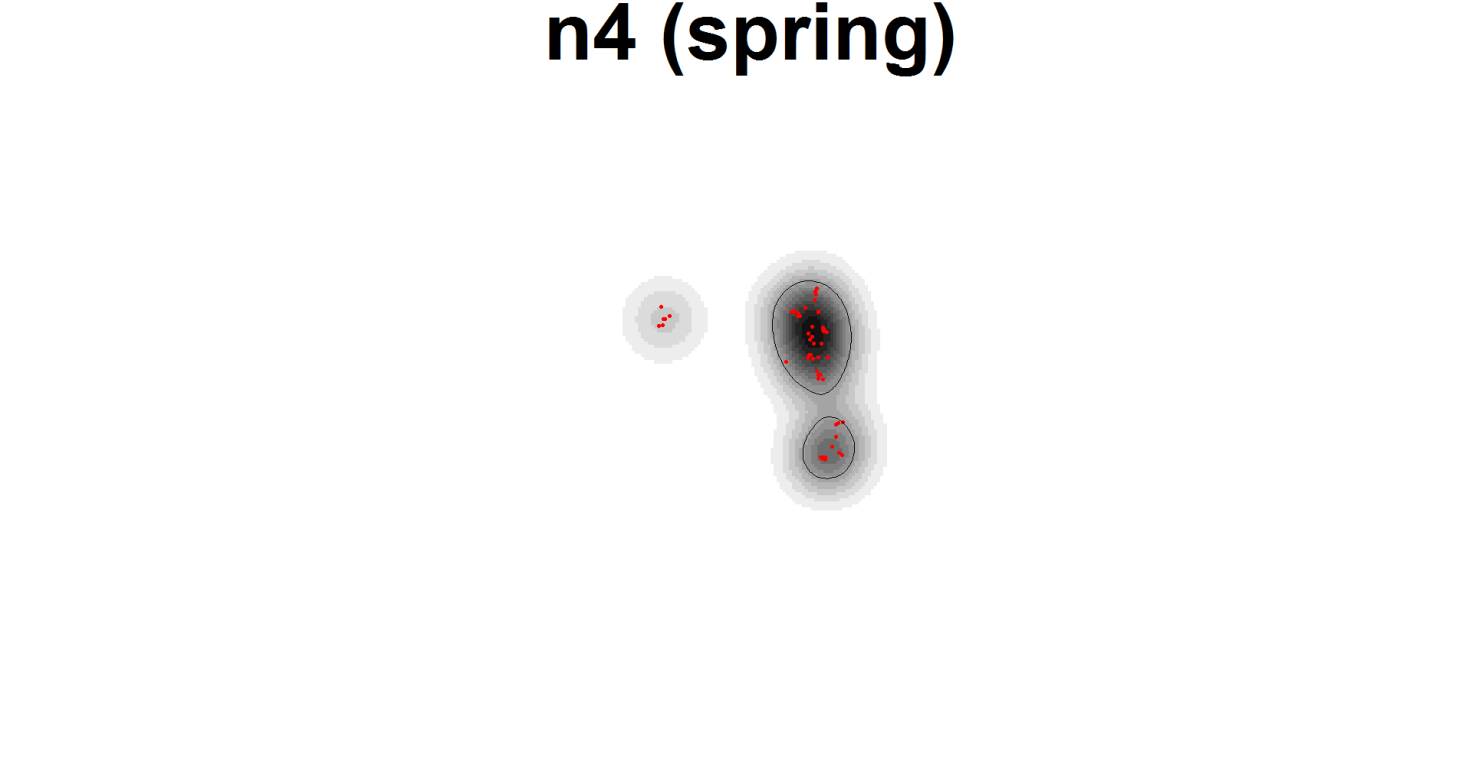

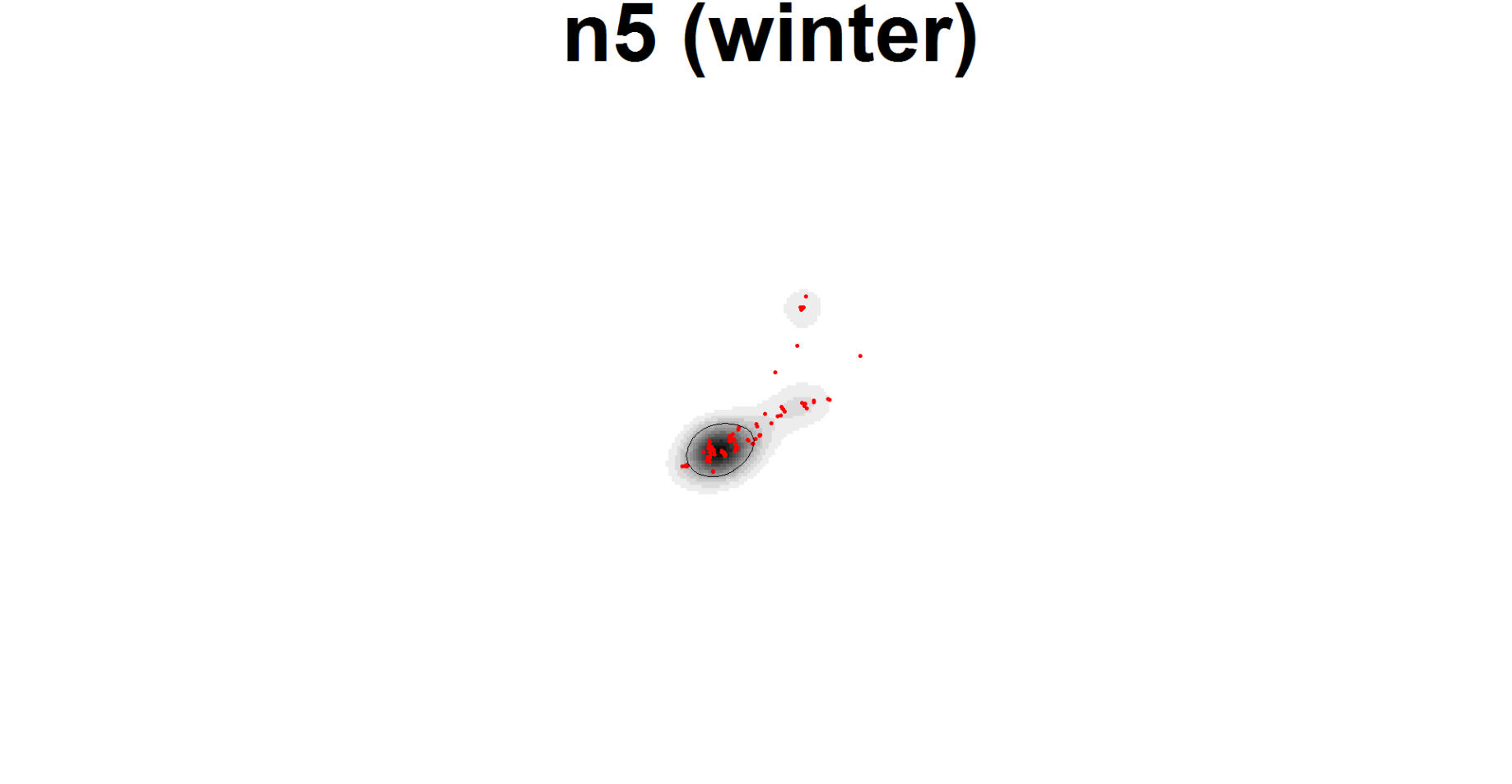

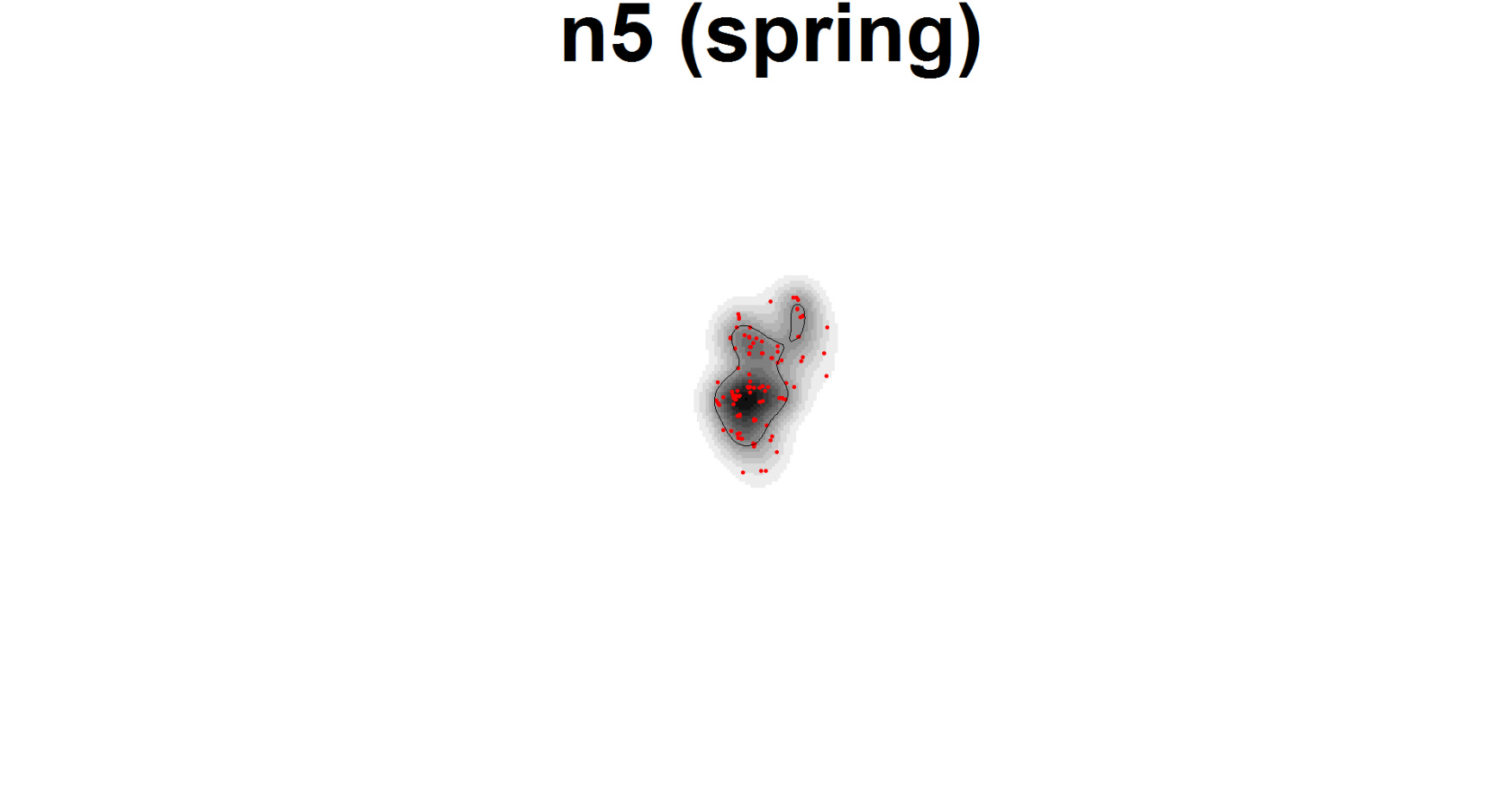

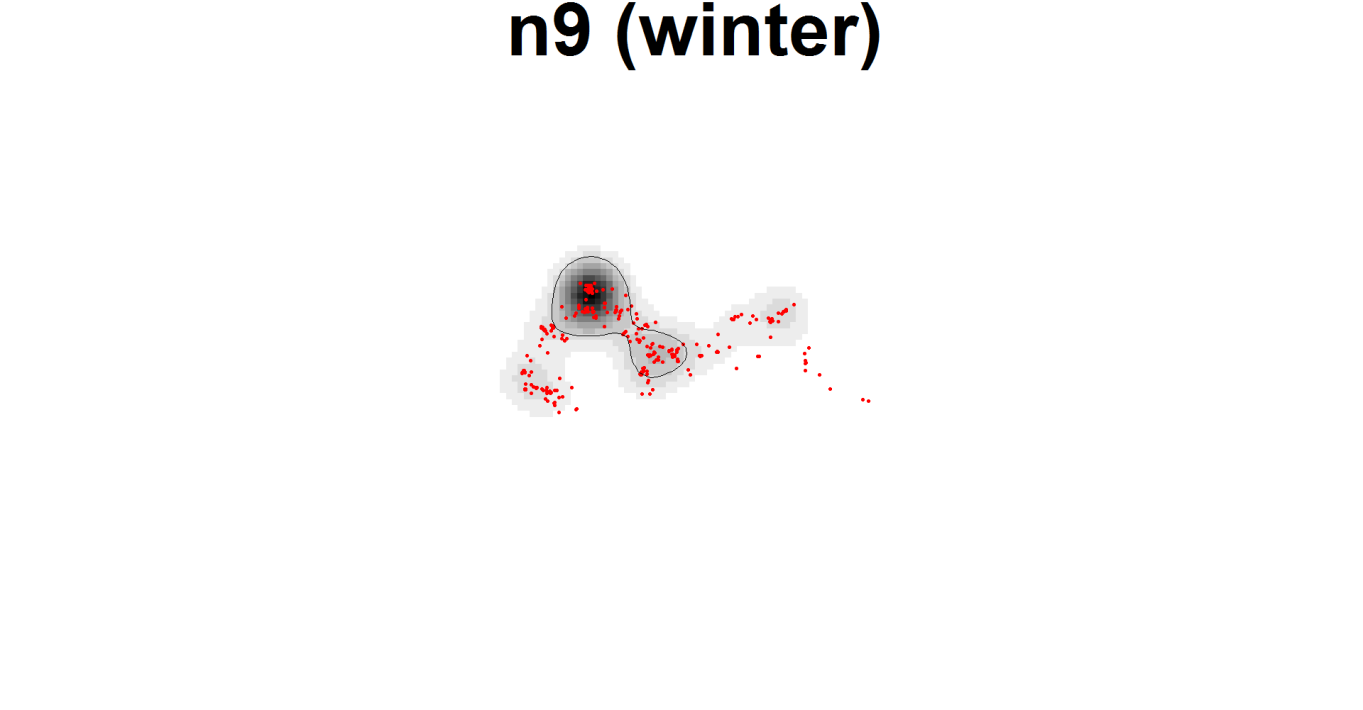


n9 (winter)


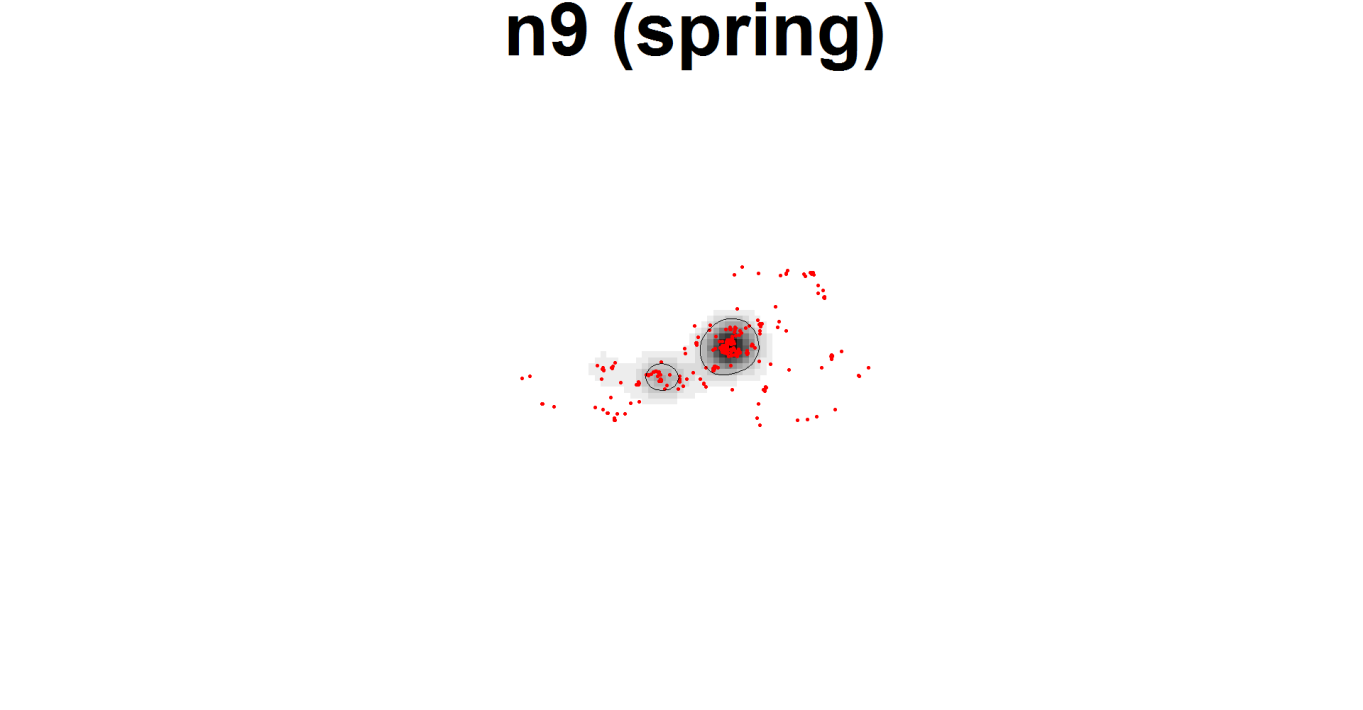


n9 (spring)


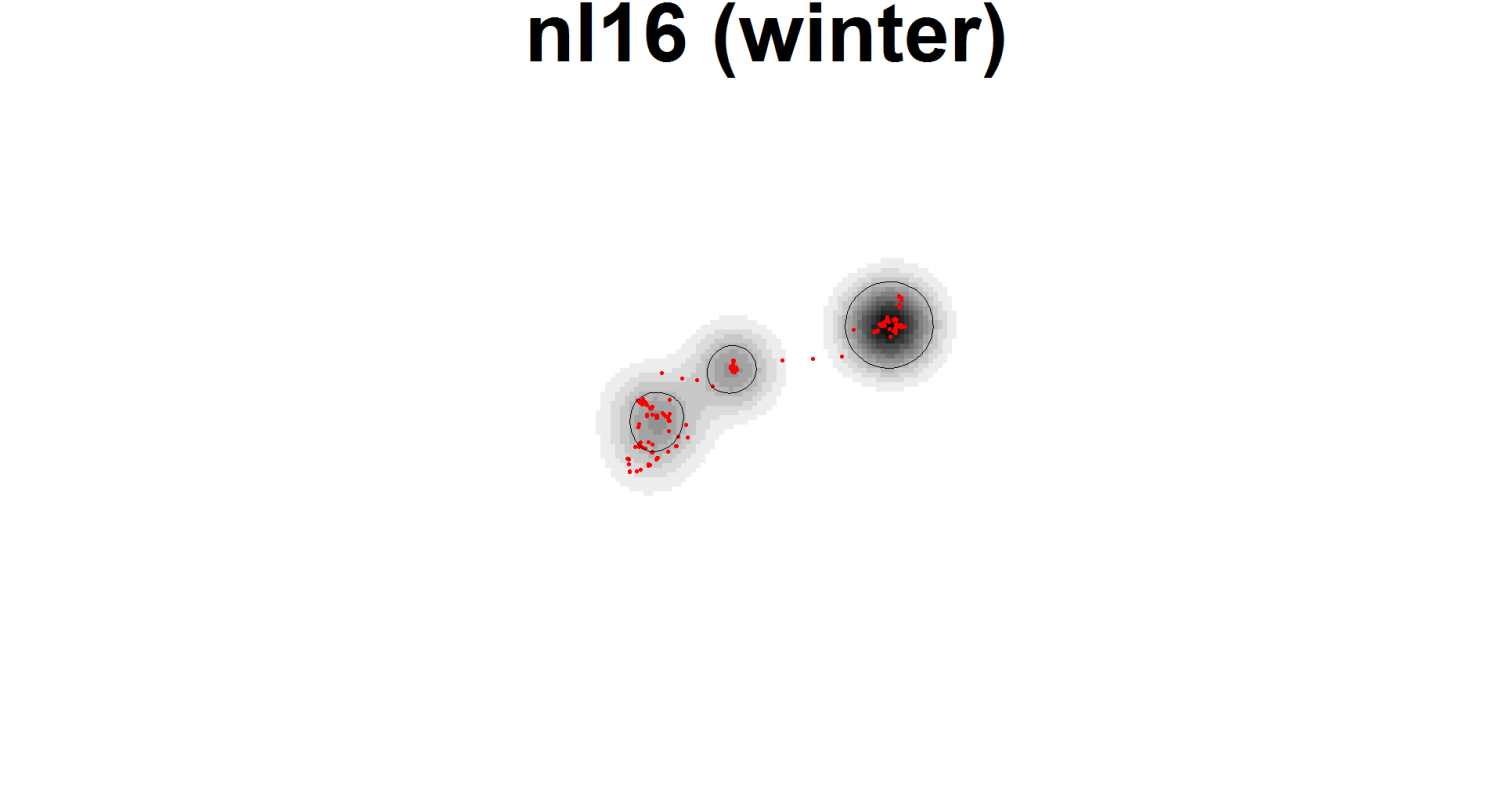


nl16 (winter)


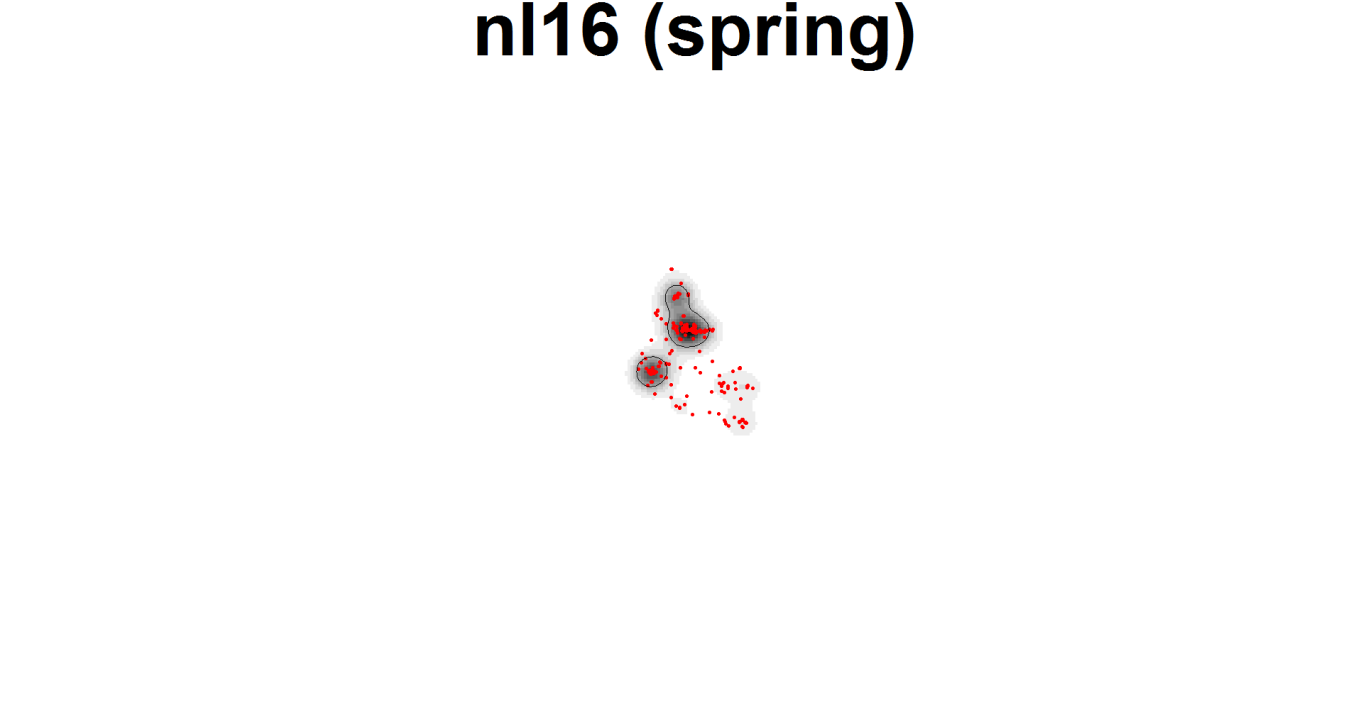


nl16 (spring)


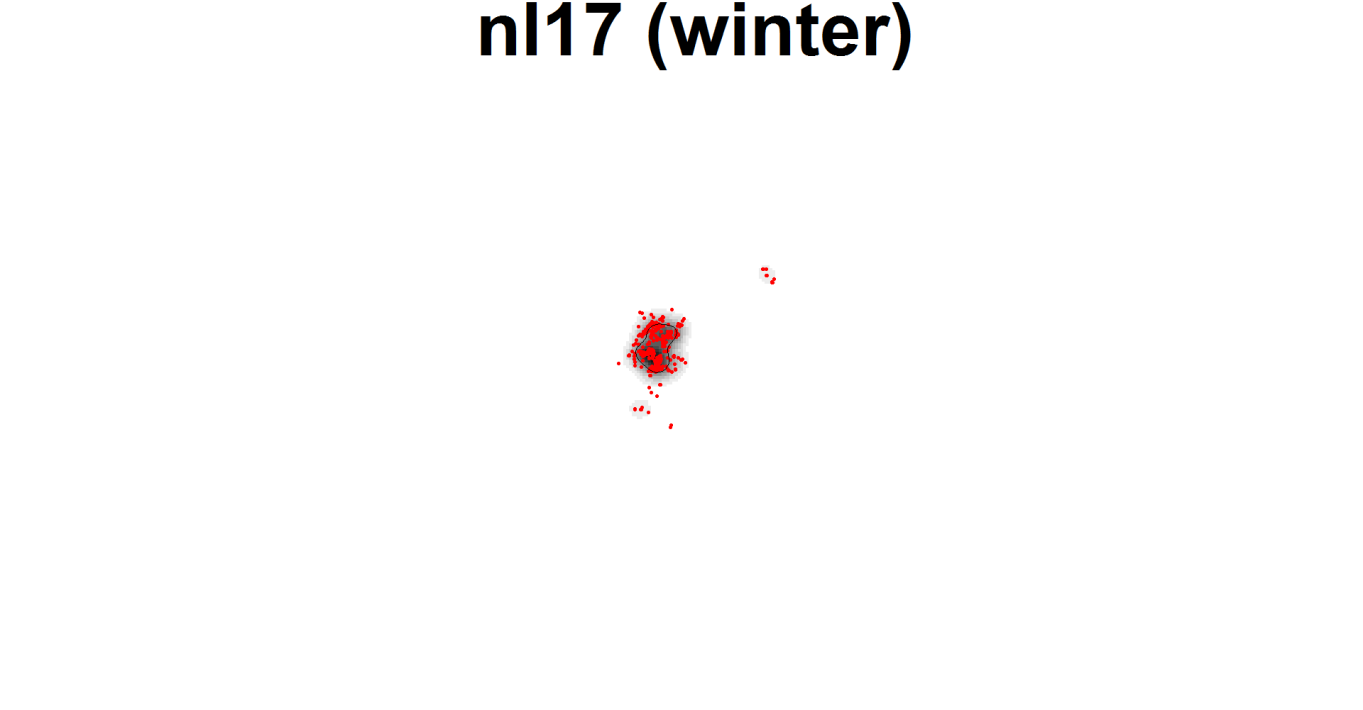


nl17 (winter)


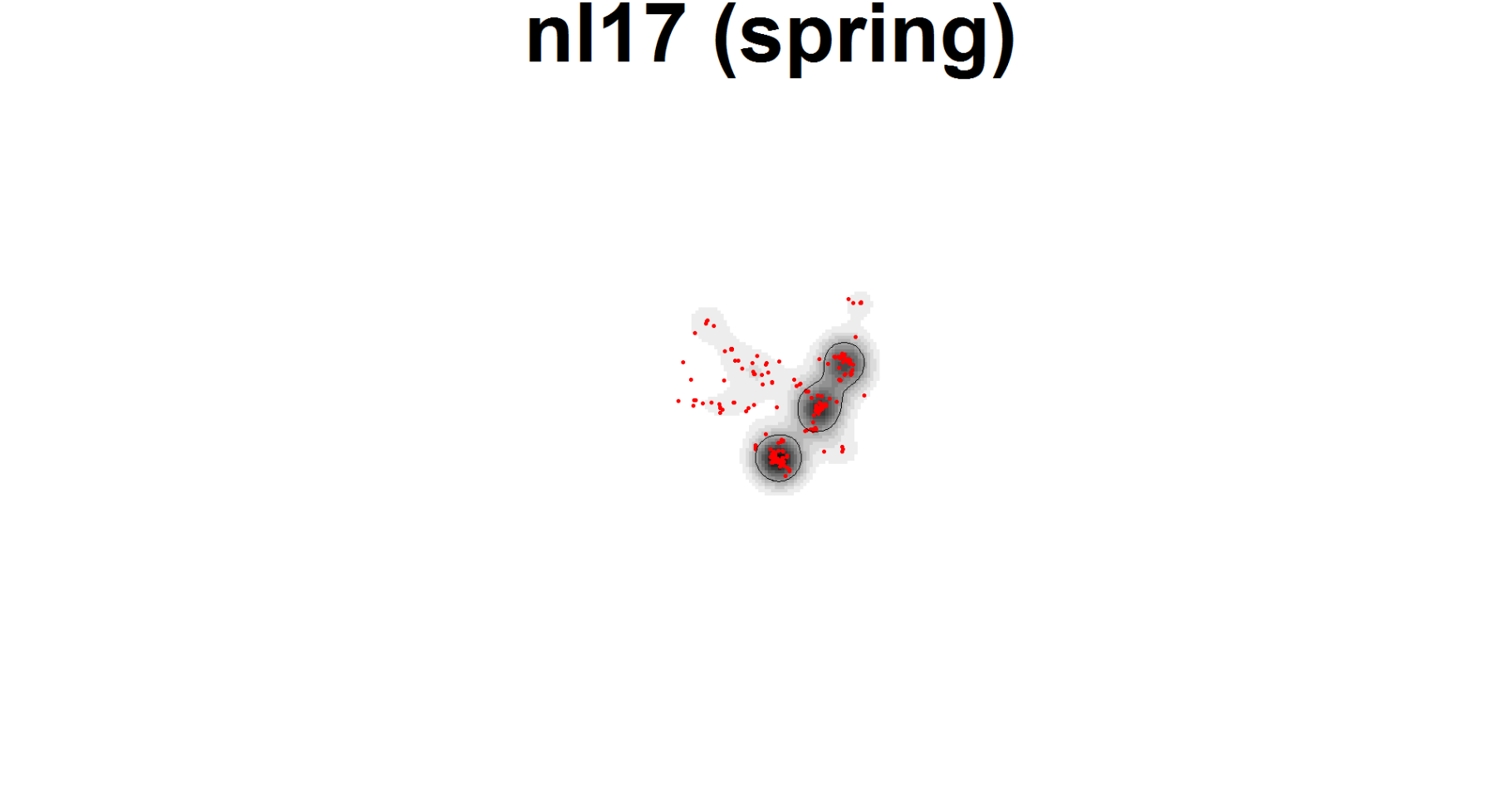


nl17 (spring)


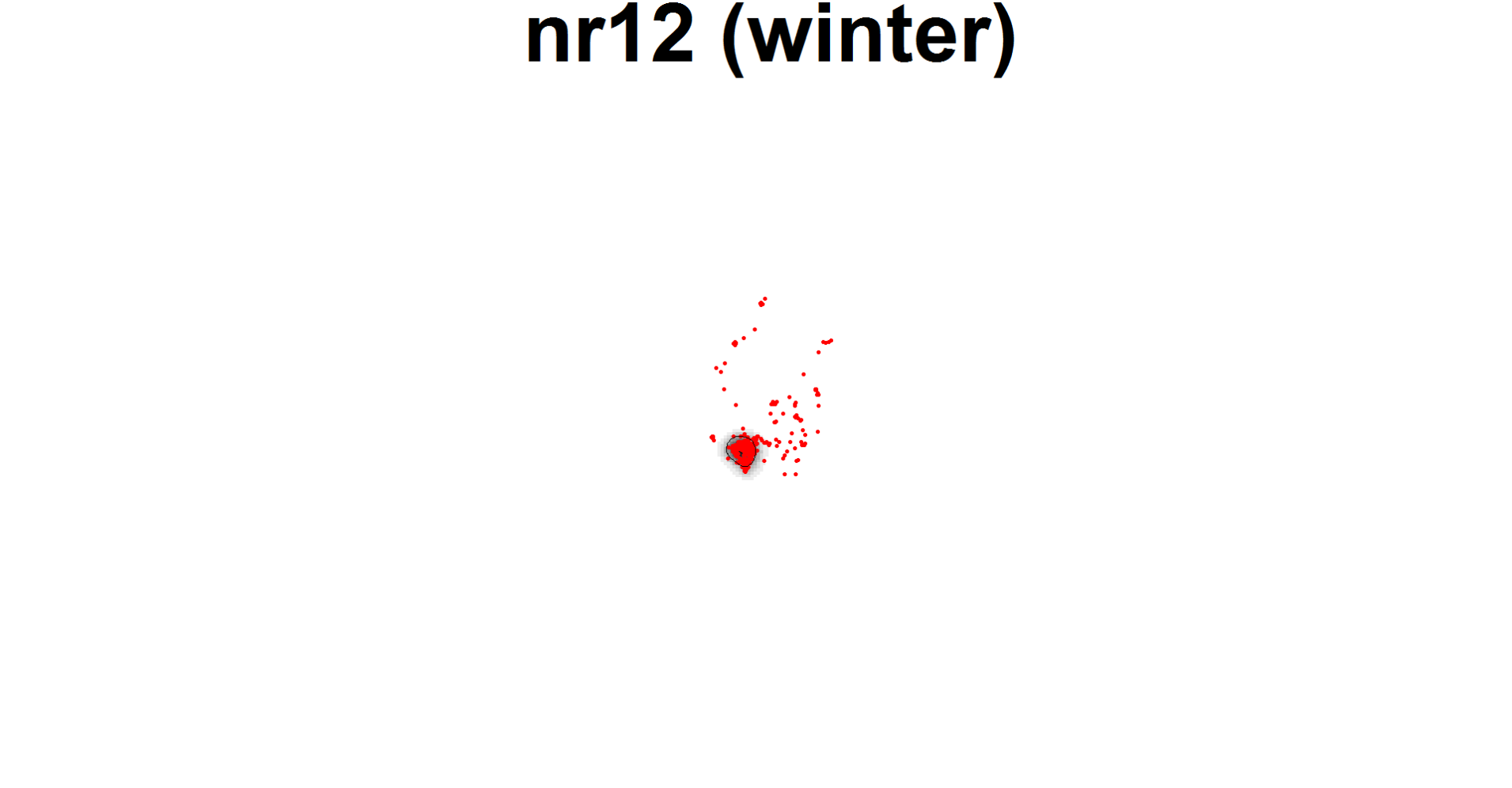


nr12 (winter)


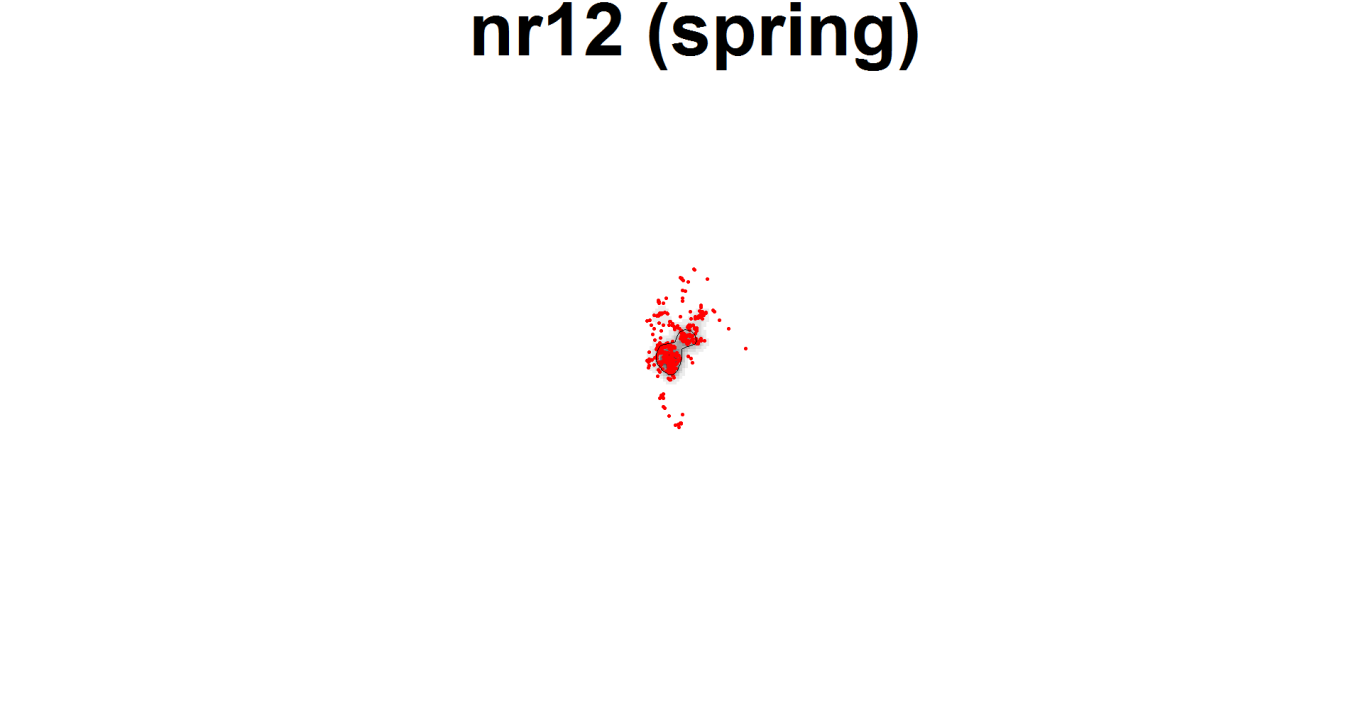


nr12 (spring)


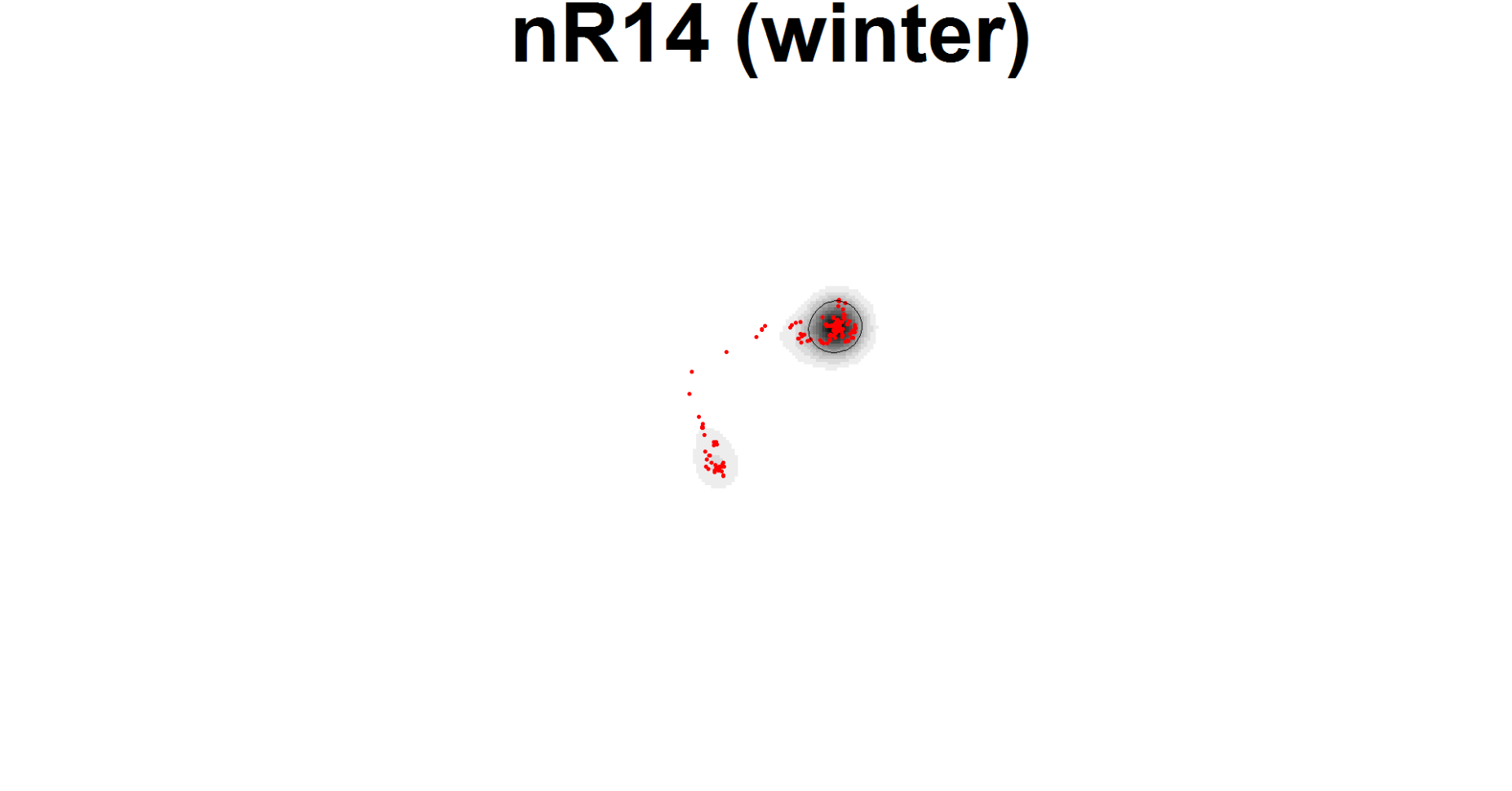


nR14 (winter)


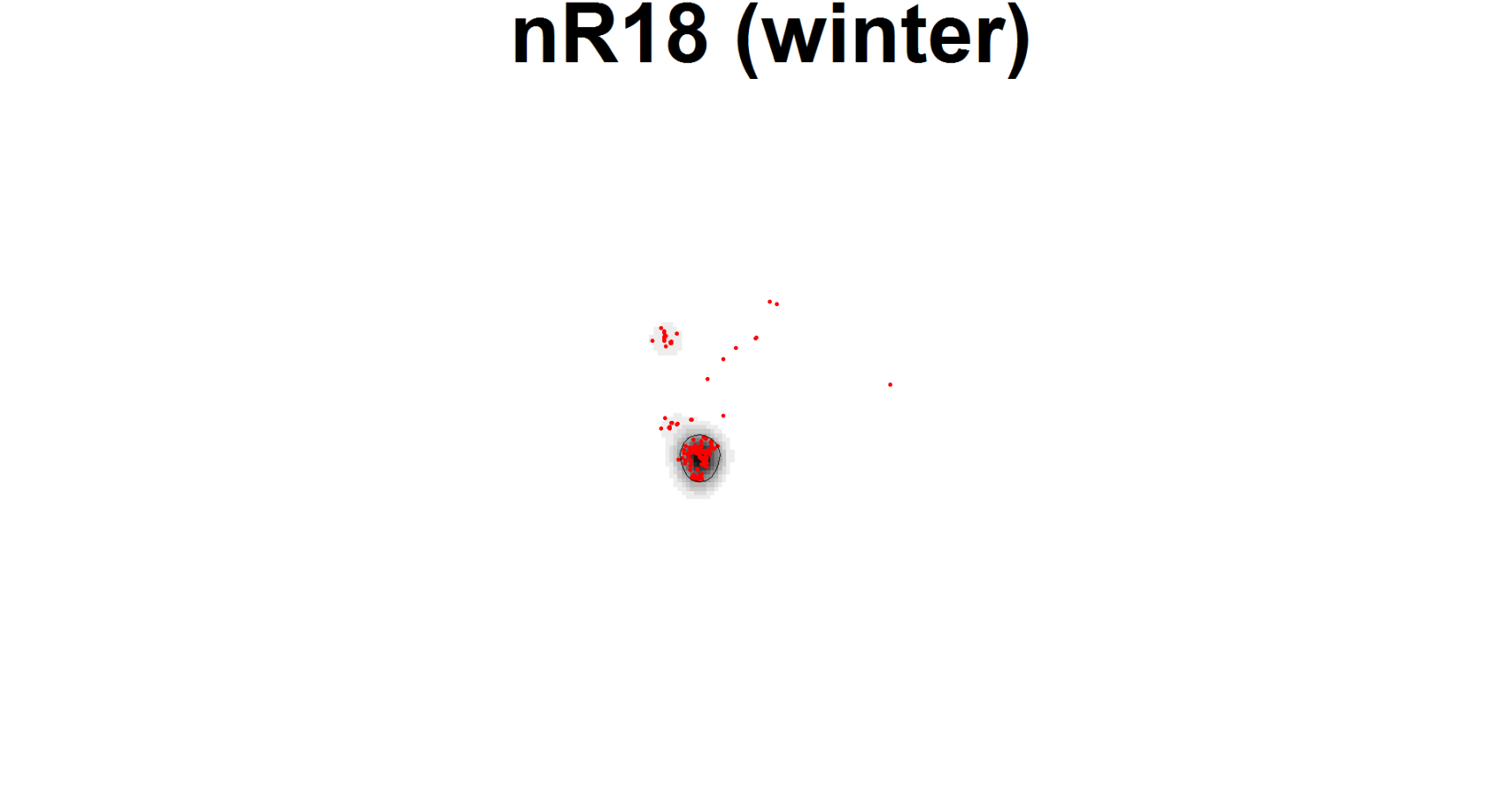

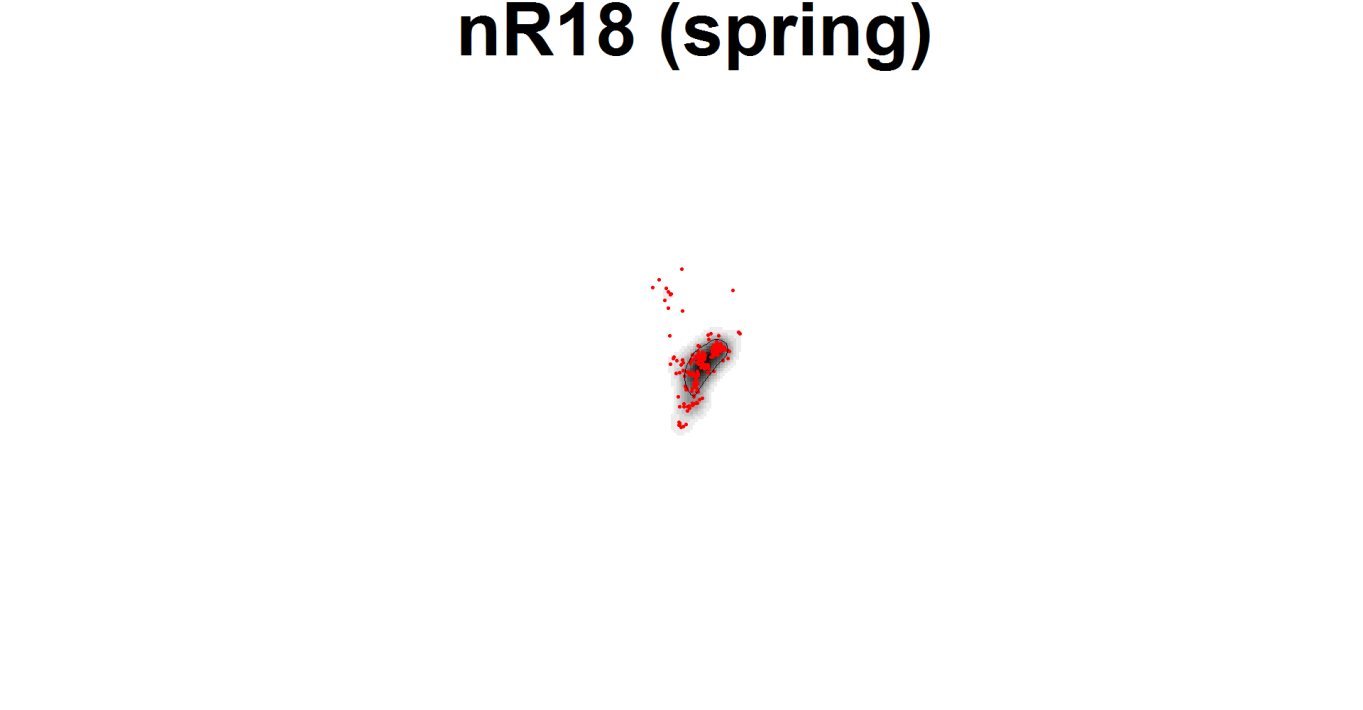

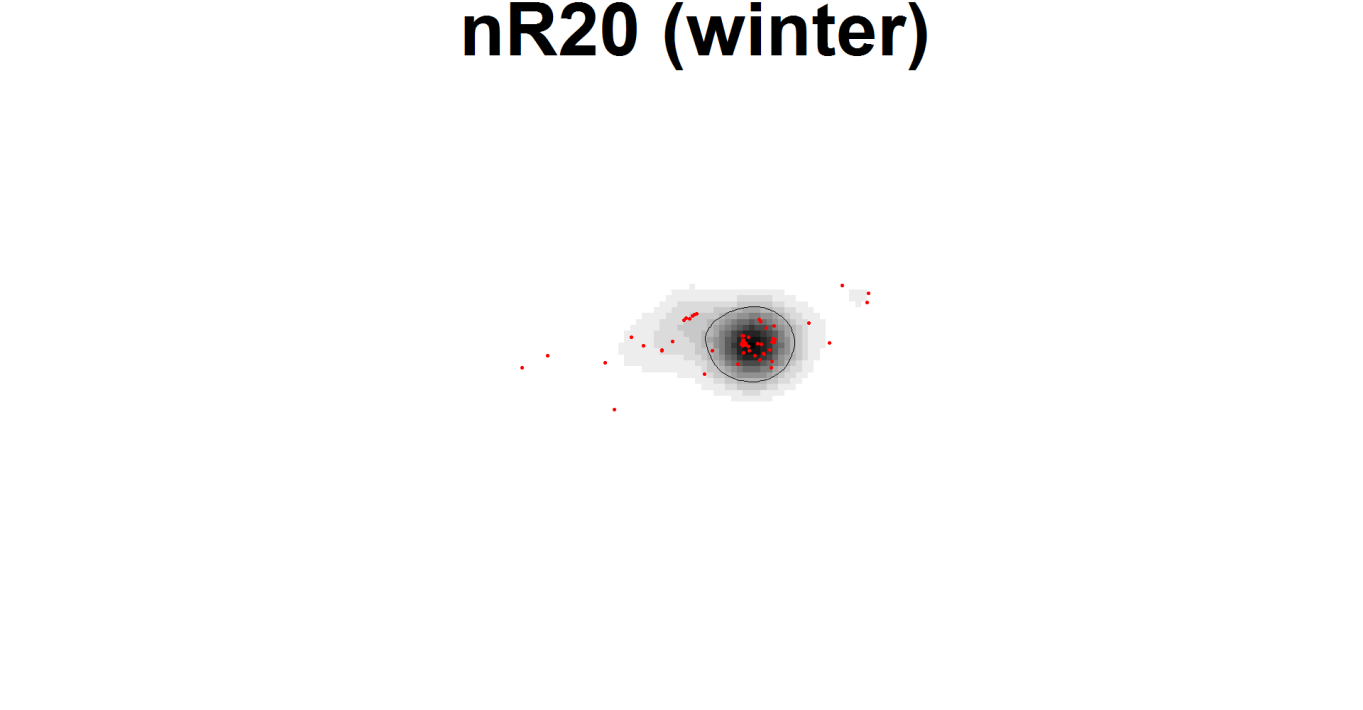


nR18 (winter)

nR18 (spring)

nR20 (winter)


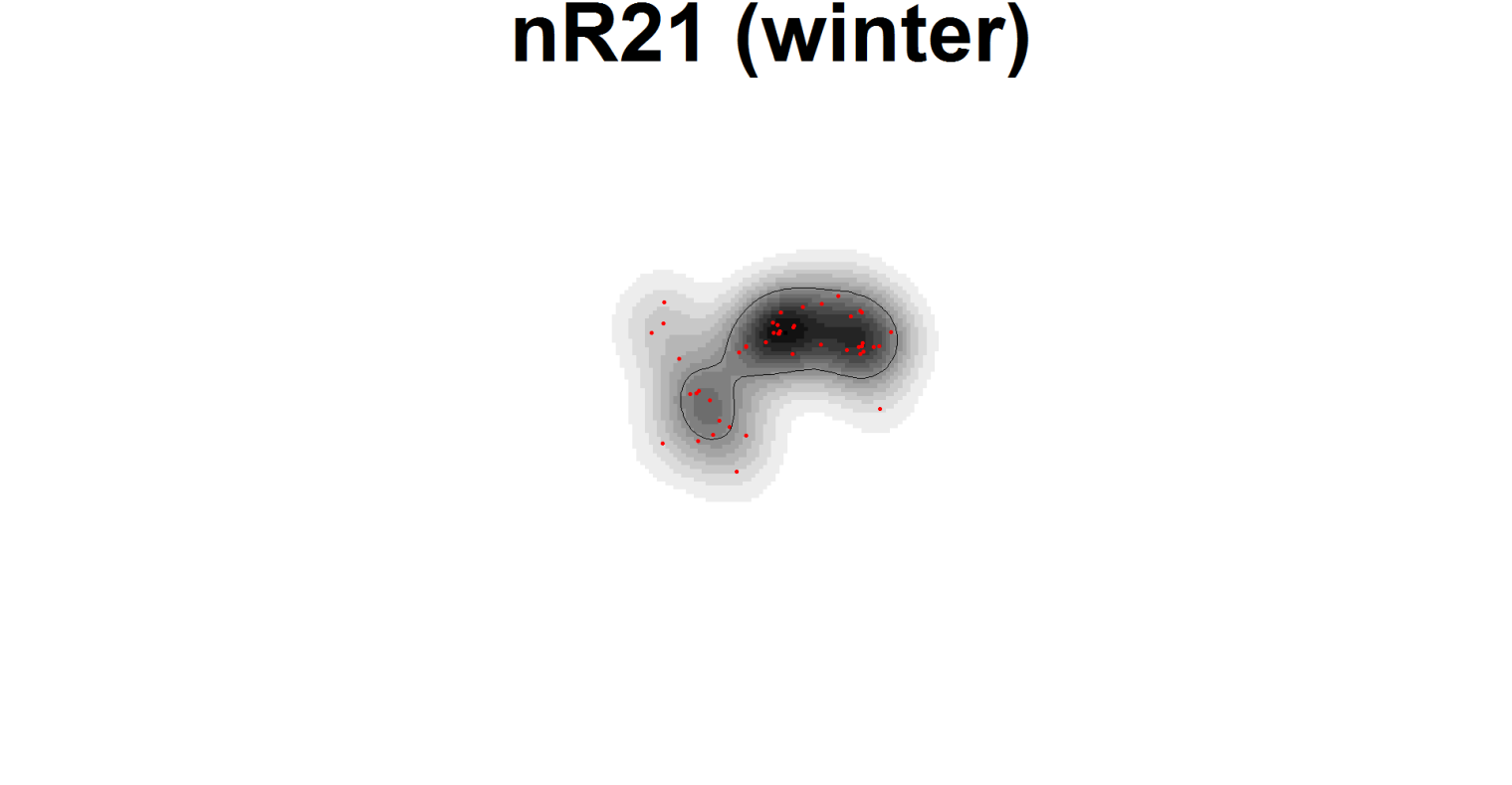

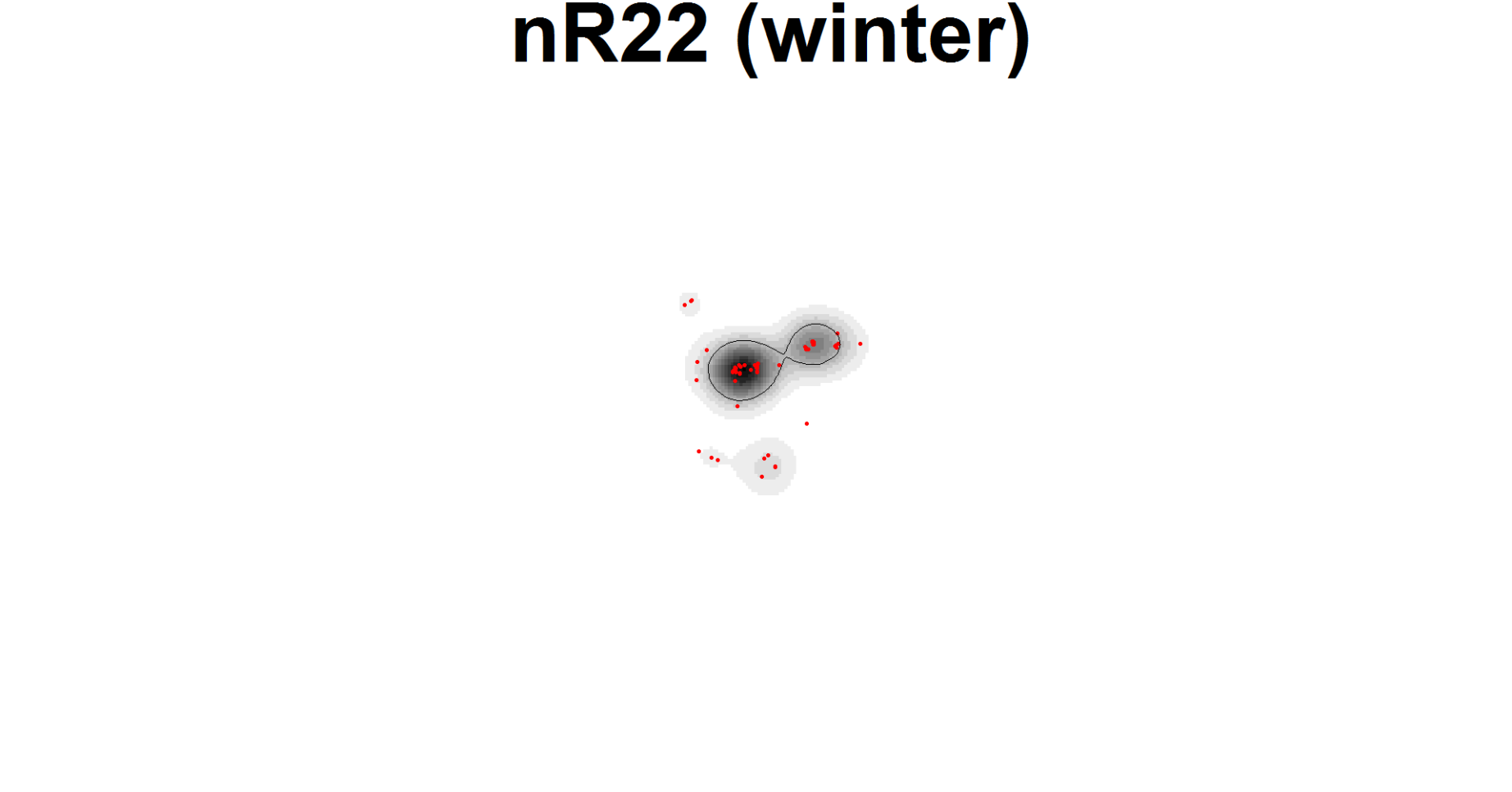

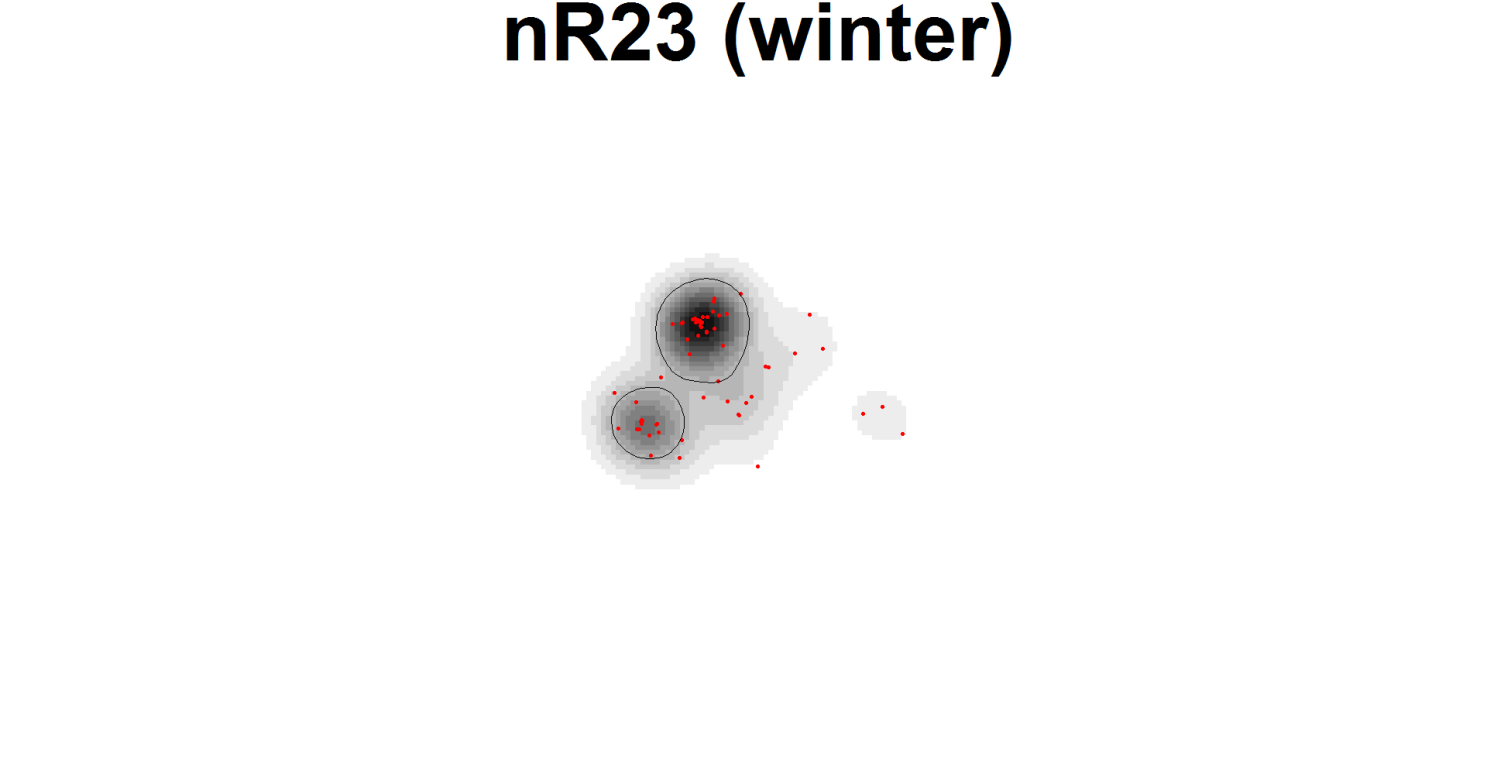


nR21 (winter)

nR22 (winter)

nR23 (winter)
