# Supplementary material for: Integrating sign surveys and telemetry data for estimating brown bear (Ursus arctos) density in the Romanian Carpathians
Source: Ecol Evol. 2017 Aug 1;7(18):7134–44. doi: 10.1002/ece3.3177 (PMC5606905; doi:10.1002/ece3.3177)
Supplement: Supplementary file 5 [file ECE3-7-7134-s005.docx]

**Appendix S5.** AIC table for candidate sets of N-mixture function of Royle (2004) for each survey season

| **Model** | **nPars** | | **AIC** | **delta** | **AICwt** | **cumltvWt** | **Pseudo- Rsq** |
| --- | --- | --- | --- | --- | --- | --- | --- |
| ***Season 1 (March-April 2011)*** | | | | | | | |
| p(Snow) abund(Conifer) | | 4 | 142.86 | 0.00 | 0.178 | 0.18 | 0.087 |
| p(Snow) abund(1) [Null model] | | 3 | 143.32 | 0.46 | 0.141 | 0.32 | 0.000 |
| p(Snow) abund(Altit+Conifer) | | 5 | 144.21 | 1.35 | 0.090 | 0.41 | 0.109 |
| p(Snow) abund(Deciduous) | | 4 | 144.28 | 1.42 | 0.087 | 0.50 | 0.038 |
| p(Snow) abund(Mixed) | | 4 | 144.79 | 1.93 | 0.068 | 0.56 | 0.019 |
| p(Snow) abund(Dominant) | | 5 | 145.04 | 2.18 | 0.060 | 0.62 | 0.081 |
| p(Snow) abund(Altit) | | 4 | 145.09 | 2.23 | 0.058 | 0.68 | 0.008 |
| p(Snow) abund(Forest) | | 4 | 145.09 | 2.24 | 0.058 | 0.74 | 0.008 |
| p(Snow) abund(GMU) | | 5 | 145.33 | 2.47 | 0.052 | 0.79 | 0.071 |
| p(Snow) abund(Dominant+Deciduous) | | 6 | 146.28 | 3.43 | 0.032 | 0.82 | 0.106 |
| p(Snow) abund(Dominant+Conifer) | | 6 | 146.36 | 3.50 | 0.031 | 0.86 | 0.104 |
| p(Snow) abund(Dominant+Mixed) | | 6 | 146.73 | 3.87 | 0.026 | 0.88 | 0.092 |
| p(Snow) abund(Dominant+Forest) | | 6 | 146.80 | 3.95 | 0.025 | 0.93 | 0.089 |
| p(Snow) abund(Mixed+Deciduous+Conifer) | | 6 | 146.81 | 3.95 | 0.025 | 0.96 | 0.089 |
| p(Snow) abund(Altit+Dominant) | | 6 | 146.91 | 4.05 | 0.023 | 0.98 | 0.085 |
| p(Snow) abund(GMU+Altit) | | 6 | 147.15 | 4.29 | 0.021 | 1.00 | 0.077 |
|  | |  |  |  |  |  |  |
| ***Season 2 (November-December 2011)*** | | | | | | | |
| p(Snow) abund(Deciduous) | | 4 | 174.94 | 0.00 | 0.218 | 0.22 | 0.142 |
| p(Snow) abund(Altit) | | 4 | 175.84 | 0.90 | 0.139 | 0.36 | 0.121 |
| p(Snow) abund(Altit+Dominant) | | 6 | 176.88 | 1.94 | 0.083 | 0.44 | 0.191 |
| p(Snow) abund(Mixed+Deciduous+Conifer) | | 6 | 177.38 | 2.43 | 0.065 | 0.50 | 0.179 |
| p(Snow) abund(Dominant+Mixed) | | 6 | 177.44 | 2.50 | 0.062 | 0.57 | 0.178 |
| p(Snow) abund(Dominant+Mixed) | | 6 | 177.44 | 2.50 | 0.062 | 0.63 | 0.178 |
| p(Snow) abund(Altit+Conifer) | | 5 | 177.70 | 2.76 | 0.055 | 0.68 | 0.124 |
| p(Snow) abund(Dominant) | | 5 | 177.77 | 2.82 | 0.053 | 0.74 | 0.122 |
| p(Snow) abund(GMU+Altit) | | 6 | 178.00 | 3.05 | 0.047 | 0.78 | 0.165 |
| p(Snow) abund(Mixed) | | 4 | 178.40 | 3.46 | 0.039 | 0.82 | 0.055 |
| p(Snow) abund(1) [Null model] | | 3 | 178.43 | 3.48 | 0.038 | 0.86 | 0.000 |
| p(Snow) abund(Dominant+Deciduous) | | 6 | 178.55 | 3.61 | 0.036 | 0.90 | 0.152 |
| p(Snow) abund(Dominant+Forest) | | 6 | 179.40 | 4.46 | 0.023 | 0.92 | 0.131 |
| p(Snow) abund(Conifer) | | 4 | 179.42 | 4.47 | 0.023 | 0.94 | 0.028 |
| p(Snow) abund(GMU) | | 5 | 179.65 | 4.70 | 0.021 | 0.96 | 0.075 |
| p(Snow) abund(Dominant+Conifer) | | 6 | 179.77 | 4.82 | 0.020 | 0.98 | 0.122 |
| p(Snow) abund(Forest) | | 4 | 180.05 | 5.10 | 0.017 | 1.00 | 0.011 |
|  | |  |  |  |  |  |  |
| ***Season 3 (March-April 2012)*** | | | | | | | |
| p(Substrate) abund(1) [Null model] | | 4 | 304.72 | 0.00 | 0.154 | 0.15 | 0.000 |
| p(Substrate) abund(Mixed) | | 5 | 305.01 | 0.29 | 0.133 | 0.29 | 0.034 |
| p(Substrate) abund(Forest) | | 5 | 305.47 | 0.75 | 0.106 | 0.39 | 0.025 |
| p(Substrate) abund(Altit) | | 5 | 305.79 | 1.07 | 0.090 | 0.48 | 0.018 |
| p(Substrate) abund(Dominant) | | 6 | 305.90 | 1.18 | 0.085 | 0.57 | 0.056 |
| p(Substrate) abund(Conifer) | | 5 | 306.48 | 1.76 | 0.064 | 0.63 | 0.004 |
| p(Substrate) abund(Deciduous) | | 5 | 306.69 | 1.97 | 0.057 | 0.69 | 0.000 |
| p(Substrate) abund(Dominant+Conifer) | | 7 | 307.30 | 2.59 | 0.042 | 0.73 | 0.067 |
| p(Substrate) abund(Dominant+Forest) | | 7 | 307.68 | 2.97 | 0.035 | 0.77 | 0.060 |
| p(Substrate) abund(Altit+Conifer) | | 6 | 307.78 | 3.06 | 0.033 | 0.80 | 0.019 |
| p(Substrate) abund(Altit+Dominant) | | 7 | 307.80 | 3.08 | 0.033 | 0.83 | 0.058 |
| p(Substrate) abund(GMU) | | 7 | 307.80 | 3.08 | 0.033 | 0.87 | 0.057 |
| p(Substrate) abund(Dominant+Deciduous) | | 7 | 307.83 | 3.11 | 0.032 | 0.90 | 0.057 |
| p(Substrate) abund(Dominant+Mixed) | | 7 | 307.89 | 3.17 | 0.032 | 0.93 | 0.056 |
| p(Substrate) abund(Mixed+Deciduous +Conifer) | | 7 | 308.24 | 3.52 | 0.026 | 0.99 | 0.049 |
| p(Substrate) abund(GMU+Altit) | | 8 | 309.73 | 5.01 | 0.013 | 1.00 | 0.059 |
